# Age effects in autobiographical memory depend on the measure

**DOI:** 10.1371/journal.pone.0259279

**Published:** 2021-10-29

**Authors:** Ali Mair, Marie Poirier, Martin A. Conway

**Affiliations:** 1 Department of Psychology, City, University of London, Northampton Square, London, United Kingdom; 2 Department of Psychology and Sport Sciences, University of Hertfordshire, Hatfield, United Kingdom; 3 Department of Psychology, University of York, Heslington, York, United Kingdom; University of St Andrews, UNITED KINGDOM

## Abstract

Studies examining age effects in autobiographical memory have produced inconsistent results. This study examined whether a set of typical autobiographical memory measures produced equivalent results in a single participant sample. Five memory tests (everyday memory, autobiographical memory from the past year, autobiographical memory from age 11–17, word-cued autobiographical memory, and word-list recall) were administered in a single sample of young and older adults. There was significant variance in the tests’ sensitivity to age: word-cued autobiographical memory produced the largest deficit in older adults, similar in magnitude to word-list recall. In contrast, older adults performed comparatively well on the other measures. The pattern of findings was broadly consistent with the results of previous investigations, suggesting that (1) the results of the different AM tasks are reliable, and (2) variable age effects in the autobiographical memory literature are at least partly due to the use of different tasks, which cannot be considered interchangeable measures of autobiographical memory ability. The results are also consistent with recent work dissociating measures of *specificity* and *detail* in autobiographical memory, and suggest that *specificity* is particularly sensitive to ageing. In contrast, *detail* is less sensitive to ageing, but is influenced by retention interval and event type. The extent to which retention interval and event type interact with age remains unclear; further research using specially designed autobiographical memory tasks could resolve this issue.

## Introduction

Retrieval from autobiographical memory (AM) is a complex process, which is widely understood to involve the interaction of several cognitive systems, but which is not yet fully understood. There are a number of different tests used to measure AM, but relatively little is known about what exactly each test measures. Studies examining the effect of ageing on AM have generally treated the various tests as interchangeable, which implies that the primary construct measured by each test is a general underlying AM ability. Converging evidence from different AM tests appears to show that this general AM ability is impaired in older adults [1–10].

Yet it is difficult to reconcile the notion of a general AM deficit in older adults with the findings of several studies in which no such impairment was observed [11–16]. Moreover, the prevalence of these null results is likely to be underestimated due to the well-established “file drawer problem” in which nonsignificant findings are less likely to be published [17, 18]. The inconsistency of these findings suggests that a more complete understanding of the age-related AM deficit requires more nuance. Previous studies have varied considerably in their methodology, and these differences could explain their inconsistent results.

One difference between AM tasks is in the way performance is measured. Measurement of AM is typically concerned with either the *specificity* of a memory [3, 9], or the amount of *detail* it contains [5, 11, 13]. *Specificity* may be rated on a scale, and refers to the degree to which a described event is situated in time and space. Thus, a memory of an event that happened only once, and that lasted less than a day, is considered to be specific. In contrast, a narrative describing repeated experiences (e.g., trips to the seaside) or an experience spanning longer than a day (e.g., a weekend away) is considered to be less specific, and consequently more “general”.

Where the amount of *detail* in an AM is the primary measure, two types of detail are of principal interest: episodic, or internal, details are those corresponding to an event situated in time (a day or less) and space. Details are counted within this category only if they pertain to the event in question. In contrast to episodic details, semantic details represent a broader category including information describing repeated or temporally extended (longer than a day) experiences, as well as decontextualised facts, which may be personally-relevant (e.g., *I am a vegetarian*, *John is my brother*) or universal (e.g., *Paris is the capital of France*). In some coding schemes, semantic details are nested within a still-broader category of external details, which also includes episodic details *not* pertaining to the event in question, as well as repetitions, metacognitive statements, and editorialising [5].

Although the qualifying criteria for specific AMs and episodic AMs are almost identical, specific AM is primarily a measure of memory search and initial retrieval, while episodic detail in AM is primarily a measure of memory elaboration. Thus, for an AM to be specific it does not need to be recalled in much detail, a participant need only recall *that* it happened. Conversely, the scoring of episodic AM is expressly concerned with the number of details that can be recalled about *what* happened. Evidence concerning the relatedness of the two measures comes from two large studies in which participants each recalled several memories. In one study, the proportion of specific memories retrieved was not correlated with the amount of episodic detail the memories contained [19], and specificity, but not detail, was positively correlated with executive control. This finding is consistent with other studies showing an association between executive function and AM specificity [7, 20]. The other study examining the relationship between specificity and detail found a medium positive correlation between the two, concluding that the two constructs are related but separable [21]. Both studies suggest that tasks measuring specificity and detail are not interchangeable; one possible reason for inconsistency in the ageing literature, therefore, is that these two measures are not equally sensitive to ageing.

Another source of difference between AM tasks is the type of cue used to elicit a memory. Previous work has shown that different types of cues differ with regard to how readily they access AMs. A distinction frequently drawn in the AM literature is that of direct versus generative retrieval [22]. Direct retrieval describes a process by which a cue leads directly to a memory without the rememberer being conscious of any intermediate processing. That is, direct retrieval appears, introspectively, to be effortless. In contrast, generative retrieval is an effortful, multi-stage process in which a cue is consciously elaborated and a retrieval strategy is engaged in order to search AM. When a candidate AM is produced it is evaluated, and if task criteria are met (e.g., *was it a one-off event*? *Did it last less than a day*?) then direct retrieval follows; if not, the generative process is reiterated until either a suitable AM is accessed or the attempt is abandoned. Previous research has shown that cueing AM with personally relevant words (e.g., friends’ names) led to direct retrieval more often than cueing with generic words [23, 24]. When cues are generic, concrete or high-imageability words appear to lead to direct retrieval more often than abstract words [25].

In earlier work, AM retrieval was shown to be faster when cue words (e.g., *jumper*) were primed by goal-derived concepts (e.g., *birthday presents*), which are thought to activate schema knowledge, compared to when they were primed by taxonomic categories (e.g., *clothing*), which are thought to activate decontextualised knowledge [26]. Faster retrieval was associated with memories that were more specific and higher in personal importance [26]. In two studies in which participants were required to generate mental images in response to verbal cues, the mental images were predominantly of AMs when the cues were emotions, goal-related concepts, routines or locations, but when cues were taxonomic categories, personality traits, or objects, participants’ mental images were more generic [27, 28]. Thus, some types of cues appear to lead readily to the retrieval of AMs even when AM retrieval is not an explicit task requirement, whereas for other types of cues the association with AM appears to be less automatic and more effortful. The heterogeneity of cues employed across previous studies of AM in ageing could contribute to inconsistent findings if, for example, either effortful or automatic retrieval processing is disproportionately affected by ageing.

The evidence outlined above suggests that there are at least two independent dimensions–measurement type and cue type–along which AM tasks can vary; however, there may be many more. The ostensibly variable age effect across tasks is not only a question of methodological interest. There are also implications for applied work, because AM tasks vary in the extent to which they reflect typical modes of remembering in the real world. For example, the requirement to retrieve a specific memory in response to a generic cue word, although a useful way to test hypotheses in a laboratory setting, bears little relation to the type of memory processing that is usually engaged outside the laboratory: when reminiscing with a friend, for instance, or when recalling how a particular problem was solved last time it was encountered. Thus, if older adults perform poorly only on less ecologically valid measures it may be a waste of resources to pursue an intervention aimed at improving performance on these measures with the expectation of improving real-world memory function. Moreover, investigation of the pattern of age effects across multiple tasks could show that impairments in certain auxiliary processes (or working-with memory processes; [29]) causes impairments in certain tasks.

This paper does not aim to provide a definitive answer to the question of whether or not older adults’ AM is impaired when compared with younger adults. Instead, the aim is to examine whether the *relative* performance of older and younger adults differs across a representative set of tasks, within a single representative sample. Thus, in the present study, a battery of AM tasks is administered, based on those used in previous work. If the different tasks converge on the same underlying construct, namely the ability to recall specific, highly contextualised personal memories, then performance across tasks should be correlated and age effects should be found across measures. On the other hand, if the tasks measure different aspects of AM, or differ in the extent to which they recruit auxiliary processes that are affected by age, then age effects should vary by task. A further aim is to explore whether the pattern of findings can offer insight into the factors contributing to the tasks’ sensitivity to ageing. Aside from its theoretical contribution, improved understanding of these commonly-used AM tasks will also be important for solving the applied issue of how to support real-world memory in older adults.

## Method

### Participants

Sample size estimation was complicated by the wide range of effect sizes across the different tasks used in previous research. Table 1 shows effect sizes for the main effect of age in tasks similar to those employed in the current study, ranging from very large to very small. For the present investigation, we ran a G*Power sensitivity analysis [30] for a 2 x 5 repeated measures ANOVA, between factors effect. We set the intended sample size at 40 (20 in each group; which is fairly typical of AM studies), with an alpha of .05 and 80% power. The analysis showed that the sample should be sufficient to detect an effect of *d* = .70, which would replicate the pattern of results shown in Table 1.

**Table 1:** Effect sizes for age effects in published AM research.

Measure	Publication	Cohen’s *d*	Main effect of age in original study?	Sample size estimate*
AM lifetime periods (childhood to past year) + list of events	Levine et al. (2002)	1.57	Yes	10
AM lifetime periods + specific questions	Piolino et al. (2006)	1.26	Yes	16
AM lifetime periods (past year only) +list of possible events	Levine et al. (2002)	0.81	Yes	32
AM word-cued	Beaman et al. (2007)	0.81	Yes	32
AM lifetime period + paper checklist of questions	Piolino et al. (2002)	0.62	Yes	52
AM lifetime periods (past week, past year, past 10 years)	Aizpurua & Koutstaal (2015)	0.22	No	392
Everyday memory	Mair et al. (2017)	0.06	No	5236

* Total sample size estimated for 80% power to detect a between-subjects main effect in 2 x 5 design

Ethical approval for the study was granted by the Psychology Department Research Ethics Committee at City, University of London (UK). All participants provided written informed consent prior to taking part.

Twenty young (age 20–31, *M* = 25.05, *SD* = 4.04; 18 female) and 20 older adults (age 66–82, *M* = 70.60, *SD* = 4.11; 17 female) participated for a payment of £20. Young adults were recruited via posters displayed around the City, University of London campus, while older adults were recruited from a panel of individuals over the age of 65, who had previously responded to a local newspaper advertisement. All participants had self-reported normal or corrected-to-normal vision and hearing. Older adults were screened for early indicators of dementia using a cut-off of 24 on the Mini Mental State Examination (MMSE [31]). Both older and younger adults completed the Geriatric Depression Scale (GDS [32]), which provided an indicator of depressive symptoms that may be associated with reduced memory specificity. To provide a measure of IQ, all participants completed the two-subscale version of the Wechsler Abbreviated Scale of Intelligence (WASI [33]) and the number of years of formal education was recorded for each participant. The results of the screening tests are presented in Table 2. Older adults were less educated than young adults but had significantly higher IQs. Both groups had similar scores on the GDS.

**Table 2:** Group depression, IQ and education scores.

Sample characteristics	Young adults		Older adults		*t*
	*M*	*SD*	*M*	*SD*	
IQ	111.50	9.06	119.45	14.47	2.08*
Education	16.85	2.54	14.11	4.38	2.41*
GDS	4.50	3.66	5.39	5.96	.56

*p < .05

### Design

The tasks employed for the present investigation involved measurement of both episodic and semantic components memory. In AM research, episodic AM refers to memories of specific events that lasted less than a day, while semantic AM is a broader category that incorporates repeated or temporally extended events and autobiographical facts (e.g., *I am the oldest in the family*; see [34]). Although any given AM will generally contain both episodic and semantic information, in the present study episodic and semantic memory were analysed separately because only three of the five tasks measured semantic recall. For episodic memory, a 2 (age: young vs. older adults) x 5 (memory test: Everyday Memory vs. Age 11–17 AM vs. Past Year AM vs. Word-Cued AM vs. Word List Recall) mixed design was employed with memory test as a repeated measures factor. Semantic memory was measured in the first three tasks only (Everyday Memory, Age 11–17 AM, Past Year AM), therefore a 2 x 3 design was employed for the investigation of semantic AM. The scoring protocols were different for each task; a description of each is presented below, and coded examples are available on the Open Science Framework project page (at https://osf.io/9wxyz/).

### Materials and procedure

Participants were interviewed individually, and each completed the same five memory tests. Tests comprised four measures of event memory (three autobiographical memory and one everyday memory) and one laboratory episodic memory task, which was intended as a benchmark for performance. The tasks measured memory for different types of experience (i.e., high or low in personal significance and distinctiveness), involved different retrieval cues (event title, time period, generic word) and different retention intervals (seconds, days, months, years). Two of the tasks specified precisely what the participant was required to recall (everyday memory, word list recall), while the others allowed participants to choose which events to describe. A comparison of selected features of each task is presented in Table 3. Tasks were administered in the same order for all participants, as follows: (1) Word List Recall; (2) Age 11–17 AM; (3) Past Year AM; (4) Word-cued AM; (5) Everyday Memory. Following this task order meant that the retrieval cues increased in specificity across the test session, which was intended to minimise the opportunity for specific cues from one task to prime retrieval of AMs in a different task. The advantages and disadvantages of this approach are discussed later.

**Table 3:** Comparison of selected features of tasks used in the current paper.

Task	AM Measure	Retention interval	Age at encoding	Cue specificity	Sampling type^1^	To-be remembered material
		Matched across groups?				
Everyday memory	Detail	Yes	No	Specific	Prospective	Personal, mundane
Past year AM	Detail	Yes	No	Generic	Retrospective	Personal, distinctive
Age 11–17 AM	Detail	No	Yes	Generic	Retrospective	Personal, distinctive
Word-cued AM	Specificity	No	No	Generic	Retrospective	Personal, distinctive
Word list free recall (episodic)	n/a	Yes	No	No cues^2^	Prospective	Non-personal

^1^ For tasks involving prospective sampling, the participant is aware, a priori, that the encountered information will be subject to a later memory test. For tasks involving retrospective sampling, the participant chooses the to-be-remembered events post-hoc, during the test session. Prospective tasks therefore allow for intentional, elaborative encoding practices, whereas retrospective tasks naturally examine memory for distinctive events that stand out enough to be selected during the test session.

^2^ Although retrieval cues were not provided, this task has some similarity to an AM task with specific cues in that the to-be-remembered material is clearly specified by the experimenter. This differs from AM tasks involving generic cues, in which the cues must be interpreted and elaborated, and available knowledge must be searched in order to find an appropriate memory to report.

#### Word list free recall

For this task participants were presented with a list of 25 unrelated common English nouns presented in lower case, in black font on a white computer screen at a rate of 5s per word. The presentation was deliberately slow in order to minimise the influence of processing speed as a confounding variable, since older adults’ processing speed is typically reduced [35]. Word characteristics were obtained from the MRC Psycholinguistic Database [36]. The selected words all had 5 letters, two syllables, concreteness ratings of between 500 and 650 and familiarity ratings of between 450 and 550 (both concreteness and familiarity scales range from 100 to 700). From the original pool of 56 words, 10 were excluded because (a) they differed by only one letter from another word in the list (e.g., *daisy*/*dairy*; one of each pair was excluded at random), (b) the word was much less common in British English than American English (e.g., *burro*; confirmed using Google Ngram Viewer 2009 corpora), (c) had different meanings in British and American English (e.g., *jelly*), or (d) pronunciation was highly irregular (e.g., *choir*). Of the remaining 46 words, 25 were randomly selected for inclusion (see OSF project page for full list: https://osf.io/9wxyz/). Immediately after presentation of the word list, participants were allowed 3 minutes to verbally recall as many words as possible, in any order. If participants indicated that they had finished the recall attempt within the 3 minutes but they had not recalled all 25 words, the timer was kept running and they could add to the recall at any point until the 3 minutes had elapsed. The score for this task was the number of words correctly recalled. This task was low in ecological validity, but presented the clearest opportunity to measure intentional memory ability, while holding constant the retention interval and to-be-remembered material.

#### Age 11–17 AM and past year AM

In this task, participants recalled one event from a temporally remote lifetime period and one event from a recent lifetime period; both events were required to be specific (i.e., not repeated events), and to have lasted a day or less. For the remote period, participants described an event that happened when the participant was aged between 11 and 17 (Age 11–17 AM), and for the recent period they described an event that had happened in the past year, but excluding the last month (Past Year AM). For Age 11–17 AM, age-at-encoding was matched in the two groups, but retention interval was not; for Past Year AM, retention interval was matched but age-at-encoding was not. To assist with the selection of an event, participants were provided with a list of example events that could have happened within the given time period (e.g., first day at high school, a birthday party, trip to the seaside, a meal in a restaurant), however they were permitted to describe an event that was not on the list. Participants were given unlimited time to describe the events in as much detail as possible, and if they described an event that did not fit the required criteria they were prompted once with a general cue (e.g., *Can you think of something that happened only once*? *Can you think of something that lasted a day or less*?). Although the contents of these memories are personally relevant, the mode of selection of a memory is uncommon in everyday life; it would be unusual for a friend to say “tell me about anything that happened within the past year”. This task is, therefore, relatively low in ecological validity, but the retrospective sampling approach is likely to produce memories for events that are relatively distinctive. Responses were audio-recorded, transcribed, and coded by the first author. Following the coding scheme developed by Levine et al. [5], each memory was divided into individual details which were classified as internal or external. Internal details were those that were specific to the event in question and included information about what happened, locations, time, and thoughts and emotions. Internal details in this task therefore reflected episodic memory retrieval, and are hereafter referred to as episodic details in order to main consistency throughout the report. External details included semantic details, unsolicited repetitions, reflections, and contemporaneous evaluation, as well as episodic details that referred to an event other than the one in question. For the purposes of the present study, only the semantic subcategory of external details was analysed. This resulted in two categories of detail–episodic and semantic–which were roughly equivalent to the episodic and semantic details coded in the Everyday Memory task.

#### Word-cued AM

For this task, participants were presented with six cue words (*happy*, *wildlife*, *excited*, *library*, *luck*, *occasion*) and were asked to describe, in as much detail as possible, a specific memory associated with the word, which lasted a day or less. Cues were presented verbally, and for each word two minutes were allowed for the production of a memory. After two minutes, if participants had not started speaking the response was recorded as zero and the next cue word was attempted. If participants began to speak within the two minutes, they were allowed to continue uninterrupted for as long as they required. No prompts were given in cases where participants described only general memories. Responses were audio-recorded, transcribed, and rated by the first author. Ratings were on a scale of 0–4, following the protocol of episodicity ratings devised by Piolino et al. [9]. The maximum rating of 4 was given to memories that described a specific instance (i.e., not a repeated event), that lasted less than a day, and contained at least one episodic detail. A score of 3 was given if the memory was as above, but lacking episodic detail. Memories of repeated or extended events situated in time and space received a rating of 2 if they contained some event detail, and 1 if they did not. A rating of 0 was applied in cases where there was an absence of memory, or only very general information was provided regarding the cue word. This task was relatively low in ecological validity compared to the other AM tasks.

#### Everyday memory

This task was based on Mair et al. [13], and involved the prospective sampling of 12 everyday events over a one-week period, two weeks prior to the test date. An event was defined as anything lasting between 30 minutes and a few hours, with a defined start and end, and which could be given a title. Participants were encouraged to sample events on at least 5 of the available days to ensure an even spread, and to record a range of different activities rather than, for example, 12 supermarket visits. No other guidance was provided. In Mair et al. the events were sampled using a wearable camera, and an accompanying diary booklet in which participants titled the event and rated the frequency, familiarity, and distinctiveness of the activity. In the present study, participants were not provided with a wearable camera, and instead were asked to write basic event details (who, what, where, when) in a diary booklet, along with the event title and ratings of event frequency (*How often do you do what you did in this event*?) and distinctiveness (*How distinctive was this event/how much does it stand out from your usual activities*?). Frequency and distinctiveness ratings were made on a scale of 1–10, with 10 denoting the most frequent and most distinctive events. Twelve events were sampled in this way. Participants were asked to fill in the form for each event as soon as possible after it took place, and to seal each form at the end of the day so that the details could not be rehearsed. Sealed forms were then brought to the test session, which took place at the university two weeks after the event sampling phase. During the test session, four of the 12 events were randomly selected by the experimenter for the participant to recall. The larger-than-required number of sampled events was intended so that participants did not know in advance which events they would be asked to recall, and were therefore less able to practise. For the recall task, the experimenter read the title of the event to the participant, who then had unlimited time to describe what happened in as much detail as possible. On the rare occasions that participants gave only general information about routines or repeated events (e.g., *I usually go to the supermarket near my house*), they were prompted once with a general cue to recall what had happened on that specific occasion (e.g., *Can you remember what happened on that specific occasion*?). No further cues were given. The ecological validity of this task is relatively high, as the to-be-remembered event is precisely specified, and this reflects a common mode of remembering in everyday life (e.g., a friend might say “tell me about when you went for lunch with Anna last week”). Responses were audio recorded, transcribed, and coded by the first author for the number of episodic and semantic details. Episodic details tended to be expressed as a sentence or a clause, and each detail constituted a piece of standalone information that directly described the experience of the event in question (e.g., *I saw a dog; I remember buying carrots*). In contrast, a semantic detail was a piece of standalone information that described something remembered, but not related to a specific event (e.g., *Frances is my sister; I always take the scenic route home*). Episodic details pertaining to different events, repetition, and other (non-memory) information reported by the participants (e.g., reflection, evaluation), were not counted. The coding categories in the Everyday Memory task and the Age 11–17 and Past Year tasks were very similar in scope, but it was not possible to apply the Levine et al. [5] coding protocol to Everyday Memory transcripts reliably because the recalled information was often described in an incompatible way. For example, in the Everyday Memory task it was common for participants to paraphrase, in considerable detail, conversations that had been had, or to describe multiple features of an object, person, location, and so on. In such cases the Levine protocol was extremely difficult to apply consistently.

#### Reliability of AM coding

All AM transcripts were coded by the first author. Three additional independent lay raters were provided with the scoring protocols for each task, as described above, and coded redacted subsets of transcripts (*n* = 6) from each of the autobiographical tasks. For the lifetime period AMs and everyday memories, raters were asked to count the total number of episodic and semantic details in each transcript, and for the word-cued AMs raters rated the memories on the 5-point scale described above. Independent raters were blind to the study hypothesis and were not informed about the age groups to which the transcripts belonged. However, the non-identifying content of the memories was not redacted and it is possible that group membership could have been inferred from the content (e.g., talking about grandchildren would have indicated the transcript belonged to an older participant). Two-way random intraclass correlations were calculated to determine the consistency between raters for each detail type in each AM task. Consistency was high for all coding systems, suggesting good agreement between raters (Everyday Memory: episodic *α* = .90, semantic *α* = .98; Age 11–17 and Past Year: episodic *α* = .95, semantic *α* = .90; Word-Cued AM: *α* = .98).

## Results

Anonymised data have been made publicly available on the Open Science Framework project page, alongside the memory coding schemes and short narrative descriptions of the memories retrieved by each participant in each task (available at https://osf.io/9wxyz/, DOI 10.17605/OSF.IO/9WXYZ).

### IQ and education

Due to between-group differences in IQ and education, we first checked whether these variables were correlated with memory performance, as this could cause difficulty interpreting any effects of age on memory measures. For memory tasks involving multiple observations (Everyday Memory, Word-Cued AM), correlations were calculated using the mean of all observations per participant. The critical alpha level was adjusted (*α* = .003) for 16 comparisons, and the correlations are presented in Table 4, with the 16 calculations of interest shown in bold. The magnitude of the correlation coefficients ranged from small to medium across the different memory measures, but none was significant at the adjusted alpha level. It is also worth noting that, in the third row, the correlations between memory measures and age group were generally larger, suggesting that age group explained more of the variance in memory measures than IQ or education. However, the correlations with age varied widely across tasks. For the remainder of the correlations there were no *a priori* hypotheses concerning the relationships between each of these variables, therefore correlations are provided only for transparency, and are not interpreted.

**Table 4:** Correlations between IQ, education, and memory measures.

			Episodic					Semantic		
	1. EDU	2. AGE	3. Everyday	4. Past year	5. 11–17	6. Word-cued	7. Word list	8. Everyday	9. Past year	10. 11–17
IQ	.32	.37	**.30**	**.17**	**-.13**	**.13**	**.22**	**.44**	**.26**	**.17**
1.		-.35	**.13**	**.01**	**.10**	**.44**	**.30**	**-.05**	**.05**	**-.24**
2.			.04	-.04	-.41	-.59	-.48	.64	.27	.28
3.				.33	.24	.23	.13	.17	.01	.11
4.					.28	.32	.43	.14	.61	.27
5.						.48	.19	.05	.07	-.05
6.							.52	-.23	.02	-.14
7.								-.13	.11	-.05
8.									.43	.33
9.										.45

*Note*. EDU = years of education, AGE = age group, Everyday = Everyday Memory, Past year = AM from the past year, 11–17 = AM from age 11–17, Word-cued = Word-Cued AM, Word list = Word List Recall. All correlations are Pearson’s *r*, except those involving the age group variable, which is categorical, therefore Spearman’s rho was calculated instead. Correlations of specific interest are printed in bold. Episodic memory measures are shaded in grey. None of the 16 correlations of interest were significant at the adjusted level of *α* = .003.

### Age effects across different memory measures

We next turn to the main hypothesis, which was that the effect of age would vary by task. We converted episodic and semantic memory scores to *z*-scores to permit examination of the relative performance of older and younger adults while minimising the impact of different scoring scales across tasks. That is, we were less interested in the raw scores on each task, and more interested in the differences between older and younger adults across tasks. The *z*-scores were calculated around the sample mean raw score for each individual measure, such that the sample mean *z*-score for each measure was 0, with scores above 0 indicating performance above the sample-average, and scores below 0 indicating performance below the sample-average. Thus, the smaller the effect of age on any given task, the closer to 0 the group *z*-score.

**Fig 1:**
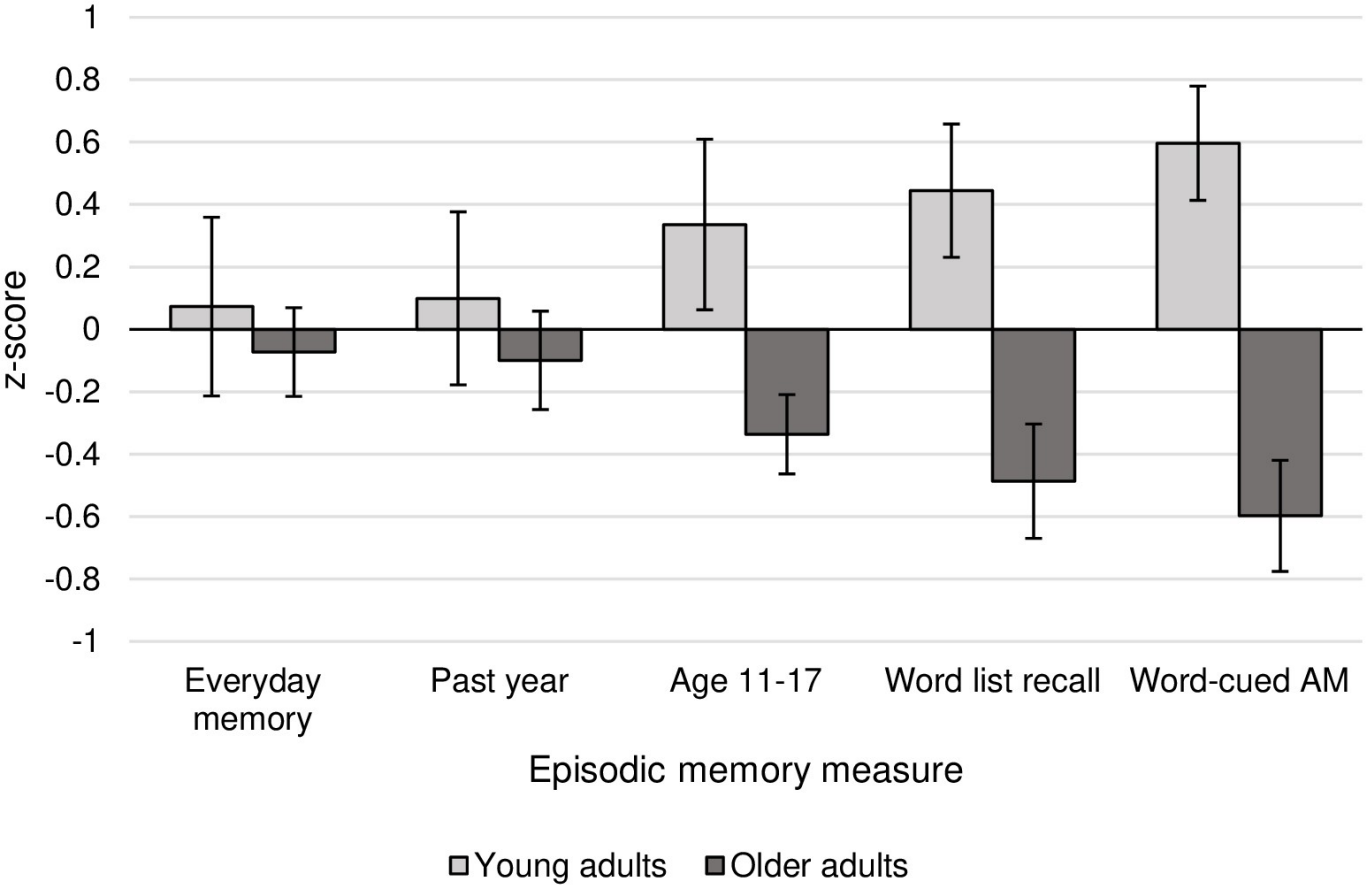
Older and younger adults’ relative performance on episodic memory measures, plotted as *z*-scores and presented in order of the magnitude of the between-group difference. Error bars represent standard error.

**Fig 2:**
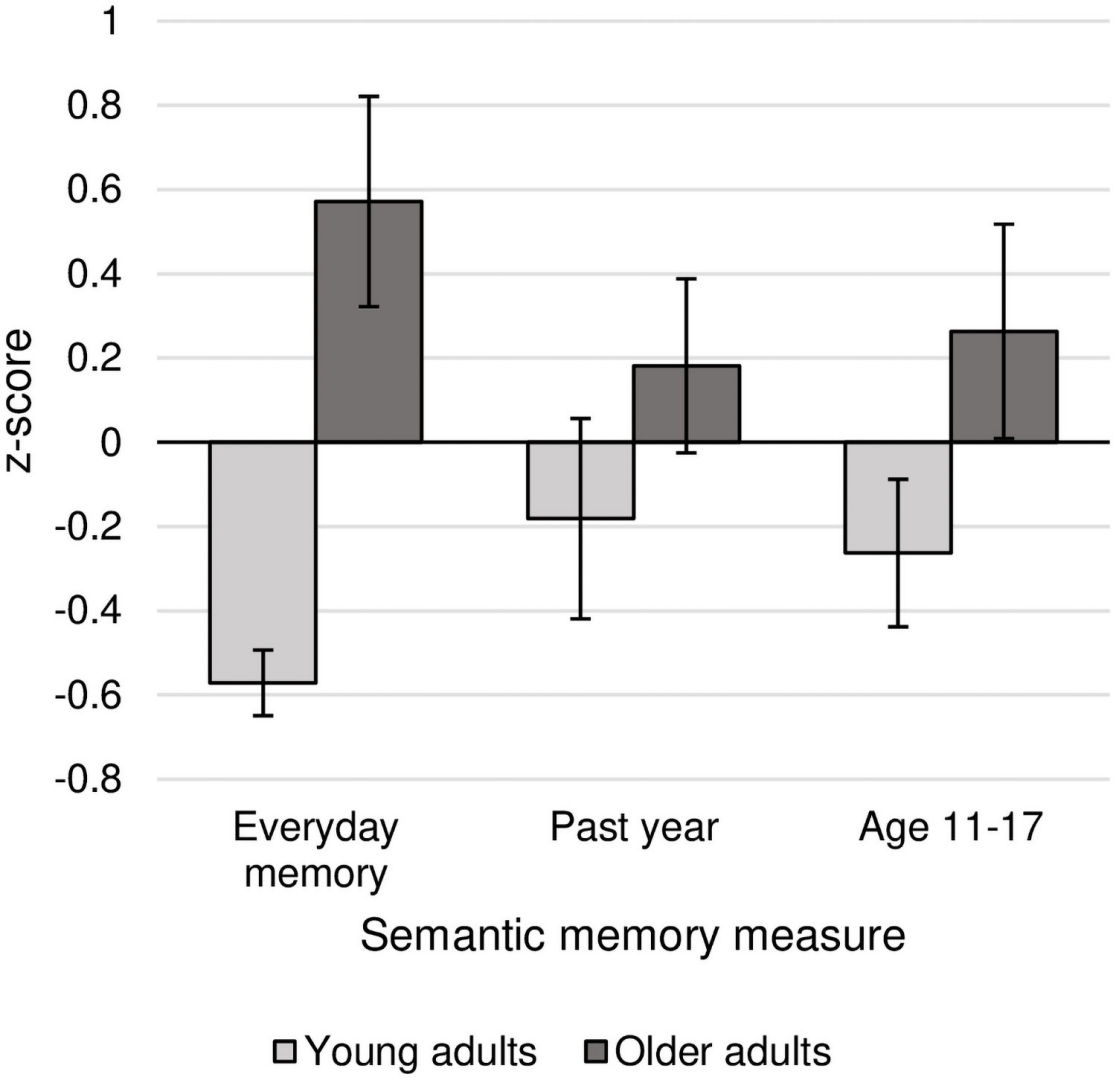
Older and younger adults’ semantic memory scores plotted as z-scores. Error bars represent standard error.

Episodic recall was measured on all five tasks, but semantic recall was only measured on three of the five (everyday memory, past year AM, and age 11–17 AM). For this reason, episodic and semantic *z*-scores were analysed separately, in a 2 (age group) x 5 (episodic score) and a 2 (age group) x 3 (semantic score) mixed ANOVA, with age group as the between-subjects factor and task as the within-subjects factor. Bayes factors were also calculated for these analyses in the JASP software package [37], using the default settings. Follow-up t-tests and Bayesian t-tests were conducted to examine age effects on each task. Bayesian t-tests were run using the default Cauchy prior width of 0.707, but specifying the directional hypotheses, based on previous research, that younger adults would outperform older adults on episodic measures, and that older adults would outperform younger adults on semantic measures. The raw scores for each of the memory measures are presented in Table 5, and the *z-*scores are plotted in Figs 1 and 2.

**Table 5:** Raw scores on each of the memory measures.

Measure	Task	Young adults		Older adults		Difference (OA–YA)
		*M*	*SD*	*M*	*SD*	
Number of episodic details	Everyday Memory	12.14	11.96	10.78	5.94	-1.36
	Past Year AM	34.10	29.03	29.45	16.49	-4.65
	Age 11–17 AM	26.20	18.17	16.20	8.45	-10.00
Number of semantic details	Everyday Memory	2.54	1.69	8.09	5.42	5.55
	Past Year AM	4.75	7.03	7.15	6.11	2.40
	Age 11–17 AM	4.85	5.95	8.85	8.65	4.00
Word count	Everyday Memory	224.05	190.42	259.06	112.99	35.01
	Past Year AM	364.70	416.19	333.25	165.51	-31.45
	Age 11–17 AM	307.05	215.60	254.15	126.59	-52.90
Episodic rating	Word-Cued AM	3.43	.57	2.60	.56	-0.83
Number of words recalled	Word List Recall	15.75	4.25	11.68	3.73	-4.07

### Episodic recall

As expected, given that the conversion of raw scores to *z*-scores centred all scores around a mean of 0, there was no main effect of task (*F*(4,152) < .01, *p* = 1.00, *η*_*p*_^*2*^ *<* .001; *BF*_*10*_ = .02), but there was a main effect of age group on episodic recall (*F*(1,31) = 10.96, *p* = .002, *η*_*p*_^*2*^ = .22; *BF*_*10*_ = 14.97). Overall, younger adults (*M*_*z*_ = .31, *SD*_*z*_ = .72) outperformed older adults (*M*_*z*_ = -.32, *SD*_*z*_ = .45) on episodic memory measures. However, this effect was qualified by a significant age group by task interaction (*F*(4,152) = 3.22, *p* = .01, *η*_*p*_^*2*^ = .08; *BF*_*10*_ = .82). This interaction was followed up with five independent t-tests, adjusted for multiple comparisons (*α* = .01), which showed significant age effects on Word-Cued AM (*t*(38) = 4.68, *p* < .0005, *BF*_*10*_ = 1052.92) and Word List Recall (*t*(38) = 3.31, *p* = .002; *BF*_*10*_ = 34.79). The age effect for Age 11–17 AM did not meet the corrected threshold for statistical significance (*t*(38) = 2.23, *p* = .03), however the corresponding Bayes factor, given the hypothesis that younger adults would outperform older adults, indicated that the data provided moderate evidence for the superior performance of young adults (*BF*_*10*_ = 4.08). This suggests that the observed data are four times more likely under this hypothesis, relative to the null hypothesis. In contrast, there was no effect of age on episodic recall in Everyday Memory (*t*(38) = .46, p = .65; *BF*_*10*_ = .44) or Past Year AM (*t*(38) = .62, p = .54, *BF*_*10*_ = .51). The small Bayes factors for these two tasks indicate that the data were insensitive, and do not constitute evidence for the null hypothesis [38]. That is, the data do not allow the conclusion that there is no difference between older and younger adults on these tasks. However, it is the differential sensitivity of the tasks to age effects which is the focus of this investigation.

Effect sizes for the effect of age in each task were converted to Cohen’s *d*, and compared to the effect sizes in previous studies employing similar tasks. The results, presented in Table 6, show that the pattern of findings observed here, within the same sample of participants, was broadly consistent with previous work conducted across multiple samples.

**Table 6:** Comparison of effect sizes for age effects in current study and previous studies employing similar tasks.

Task	Effect size (d)	
	Current study	Previous studies
Everyday Memory	0.14	0.06 ^a^
Past Year AM	0.20	0.22–0.81 ^b^
Age 11–17 AM	0.71	0.62–1.57 ^c^
Word-Cued AM	1.48	0.81–1.29 ^d^
Word List Recall	1.01	0.79–1.45 ^e^

^a^[13];

^b^ [5, 11],

^c^ [8, 9];

^d^ [2, 39, 40],

^e^ [41–43]

### Semantic recall

There was no main effect of task on semantic recall (*F*(2,76) < .001, *p* = 1.00, *η*_*p*_^*2*^ *<* .001, *BF*_*10*_ = .08). As above, this was expected due to the standardisation of scores across tasks by converting them to *z*-scores. There was a main effect of age group (*F*(1,38) = 9.25, *p* = .004, *η*_*p*_^*2*^ = .20; *BF*_*10*_ = 9.25), which showed that older adults (*M*_*z*_ = .34, *SD*_*z*_ = .84) recalled more semantic details than younger adults (*M*_*z*_ = -.34, *SD*_*z*_ = .53), consistent with findings from several previous studies [5, 11, 13]. The age by task interaction was not significant (*F*(2,76) = 2.96, *p* = .06, *η*_*p*_^*2*^ = .07; *BF*_*10*_ = .97) and consequently frequentist follow-up t-tests were not conducted. However, Bayesian t-tests compared the strength of evidence for an age effect on semantic recall in each task. There was strong evidence for older adults’ increased semantic recall in Everyday Memory (*BF*_*10*_ = 462.64), but inconclusive evidence of increased semantic recall in Past Year AM (*BF*_*10*_ = .88) and Age 11–17 AM (*BF*_*10*_ = 1.79), suggesting that these tasks were less sensitive to age differences in semantic AM. The *z*-scores for semantic recall are presented in Fig 2.

### Further exploratory analyses

In the analyses presented above the magnitude of age effect was shown to vary across tasks. There are several potential explanations for this finding, and since the richness of our dataset permitted further exploration, we performed a series of post-hoc exploratory analyses in order to investigate possible reasons for our main finding. Due to the length of the exploratory analyses only a summary is presented here; full details are presented in S1 File. In the analyses that follow we present the results of statistical tests, but due to the exploratory nature of the work these are intended primarily as a means of describing the data more fully (e.g., by providing comparative information on the strength of observed relationships). Further research would be needed to confirm these findings.

#### Retention interval

One major difference between tasks was the retention interval at which memory was measured. To examine whether retention interval was responsible for the observed age effects we looked first at Word-Cued AM. Where possible, memories retrieved in this task were dated by the experimenter, using the content of the memory to assign an approximate age where possible. Four retention interval bins were established (1 = 0–5 years, 2 = 6–10 years, 3 = 11–20 years, 4 = >20 years), to which 210 responses were assigned. The remaining 30 responses (8 young adult, 22 older adult) could not be dated. The binned mean retention interval was calculated for each group, and showed that memories retrieved by older adults (*M* = 2.24, *SD* = 1.01) were significantly older than those retrieved by young adults (*M* = 1.32, *SD* = .35; *t*(38) = 3.85, *p* < .0005). We then analysed the episodicity of only those memories assigned to the first bin, which were those from the past five years. Three older adults were excluded because they did not recall any memories from this period. For the remaining participants, young adults’ memories (*M* = 3.57, *SD* = .50) were still more episodic than older adults’ memories (*M* = 3.03, *SD* = .47; *t*(35) = 3.36, *p* = .002). We also examined the effect of retention interval by directly comparing Past Year AM and Age 11–17 AM, for which the instructions and coding protocol were identical. A 2 (age group) x 2 (task) ANOVA showed an effect of retention interval, in which Past Year AMs were recalled in greater episodic detail (*F*(1,38) = 7.71, *p* = .008, *η*^*2*^ = .17). There was no significant interaction with age (*F*(1,38) = .49, *p* = .49, *η*^*2*^ = .01), despite retention intervals being matched between groups in Past Year AM but much longer for older adults than for young adults in Age 11–17 AM.

#### Executive function

We used the Matrix Reasoning subtest of the WASI as a proxy measure of executive function, and calculated the correlation between raw unstandardised scores and each episodic memory measure (see Table 7). Matrix Reasoning scores were significantly positively correlated with episodic recall in Word-Cued AM and Word List Recall, but not in Age 11–17 AM, Past Year AM, or Everyday Memory. However, these correlations were confounded by a significant between-group difference in Matrix Reasoning scores, in which young adults (*M* = 29.15, *SD* = 2.50) outperformed older adults (*M* = 22.65, *SD* = 6.41; *t*(38) = 4.23, *p* < .0005). Thus, we also calculated the same five correlations within each age group. In older adults, we observed the same pattern as above, but in young adults none of the correlations was significant, and except for Word List Recall, coefficients were negative. Depressive symptoms, as measured by the GDS, were also negatively correlated with executive function, and this relationship was stronger in older adults than in young adults. However, GDS was not correlated with any measure of episodic recall.

**Table 7:** Correlations between Matrix Reasoning scores, each memory measure, and depression.

	Overall	Older adults	Young adults
Word list recall	.53*	.46*	.29
Word-cued AM	.59*	.61*	-.10
Age 11–17	.21	.23	-.20
Past Year	.15	.33	-.12
Everyday memory	.17	.28	-.12
GDS	-.44*	-.51*	-.34

* p < .05;

GDS = Geriatric Depression Scale.

#### Event type

Finally, we asked whether the different AM tasks led to retrieval of different types of events, which could plausibly differ in their memorability. We began with Everyday Memory, for which participants had prospectively provided subjective ratings of frequency and distinctiveness for each event. We calculated hierarchical correlations between frequency, distinctiveness, and episodic recall by calculating within-subject correlation coefficients, averaging the coefficients across the sample, and running one-sample t-tests to check whether the coefficients were significantly different from zero. The results showed that event frequency was negatively correlated with episodic recall (*t*(39) = -6.10, *p* < .0005), and event distinctiveness was positively correlated with episodic recall (*t*(38) = 5.45, *p* < .0005). Frequency and distinctiveness were negatively correlated (*t*(38) = -10.80, *p* < .0005). The pattern of correlations was the same in each age group; overall frequency (young: *M* = 4.40, *SD* = 1.17; older: *M* = 4.90, *SD* = 1.68) and distinctiveness ratings (young: *M* = 6.16, *SD* = 1.21; older: *M* = 5.42, *SD* = 2.40) did not differ between groups (frequency: *t*(38) = -1.10, *p* = .28); distinctiveness: *t*(38) = 1.24, *p* = .22). Thus, events that were less frequently experienced were more distinctive, and were better-remembered.

Equivalent ratings for the other AM tasks were not collected because the events were retrospectively sampled, however the contents of the retrieved memories were explored using the Linguistic Inquiry and Word Count programme (LIWC [44]). LIWC is textual analysis software that reads raw texts and categorises the words in relation to an inbuilt dictionary of psychological themes, narrative styles, and parts of speech, which have been shown to correlate with various psychological constructs [45]. In the absence of any theoretical framework to guide the application of LIWC to the comparison of AMs, we searched for any variables that might shed light on the content of the memories. Six clusters of variables were selected (topic, pronouns, temporal focus, themes, emotion, and social), representing 20 out of 93 total categories. The full list of included variables can be found in S5 Fig in S1 File. The full LIWC output for each task can be found on the OSF project page (DOI 10.17605/OSF.IO/9WXYZ, https://osf.io/9wxyz/). Due to the large number of variables (20 categories, 2 age groups, and 4 memory tests), we did not perform statistical analyses on these data. Instead, we plotted the percentage of words belonging to each category and visually inspected the plots. The plots are available in S5 Fig in S1 File. Patterns of word usage were similar in each age group, and varied across tasks. Age 11–17 AMs contained a high proportion of words related to work and education, and first-person singular pronouns (I, me, mine). Past Year AMs contained a high proportion of leisure words, and first-person plural pronouns (we, us, our). Word-Cued AMs were relatively high in positive emotion, but low in social words (e.g., mate, talk, they, child), whereas Everyday Memories were relatively low in emotion but high in social words, and made more references to money (e.g., buy, sale, shop). Young adults’ Everyday Memories contained a high proportion of words relating to work, while older adults’ Everyday Memories were higher in leisure words. Thus, different tasks appeared to lead to retrieval of different kinds of events, and these differences could plausibly account for differences in memory performance. These analyses are explored in greater detail in the S1 File.

## Discussion

The findings above show that various tasks typically used to measure AM are differentially sensitive to ageing. The relative performance of young and older adults on each of the five memory measures was consistent with previous studies, and the magnitude of the age effects in this study were fairly typical of each task, according to the published literature (e.g., [2, 5, 8, 9, 11, 13, 39–43]). This suggests that (1) AM tasks produce reliable results across different samples, and (2) inconsistencies in the ageing AM literature are at least partly due to the use of different tasks to measure AM. Although each of these different tasks measures some element of naturalistic memory retrieval, they cannot be considered interchangeable measures of AM ability.

In the exploratory analysis we questioned whether the variable age effect might be explained by differences in retention interval, because the AM tasks with the largest age effects appeared to be those in which older adults recalled memories at longer retention intervals than young adults. This biases the task towards young adults’ superior performance because memories tend to become “semanticised” as they age: over time, or with repeated rehearsal, episodic detail is lost and semantic detail either increases or becomes more prominent as a proportion of total detail [46–50]. This “semanticisation” is also characteristic of the age effect in AM (e.g., [5]), meaning effects of participant age are confounded by effects of memory age. In the present study, participants recalled more episodic details about memories from the past year than those from adolescence, consistent with the notion that episodic detail is usually lost over time. Moreover, the Age 11–17 task was more sensitive to age than was the Past Year AM task, suggesting that when all else is equal, longer retention intervals for older adults may negatively impact performance. In the Word-Cued AM task, no time period was specified, and here older adults’ memories were significantly older than young adults’ memories, and their performance was correspondingly poor. However, older adults’ Word-Cued AM performance was still comparatively poor when the analysis was limited to only the most recent memories, therefore retention interval could not be responsible for the age effect on this task. In addition, older adults’ Word List Recall was also relatively poor, even though retention interval was short and matched across groups. Thus, while there was some evidence that longer retention intervals might contribute to poor AM performance in older adults, the pattern of age effects could not be explained entirely by retention interval differences.

Our exploratory analyses also suggested that the AM tasks varied in their relation to executive function performance. Both AM specificity (in the Word-Cued AM task) and Word List Recall were positively correlated with executive function, while Everyday Memory, Age 11–17 AM, and Past Year AM were less clearly related. This pattern is consistent with previous work showing that AM specificity, but not detail, is associated with executive control [19]. The dissociation of specificity and detail in AM parallels an earlier distinction between memory search/construction and elaboration processes, which have been shown to rely on distinct patterns of brain activity [51, 52]. AM specificity might therefore be characterised as a measure of search and initial memory construction (remembering *that* something happened), which is associated with executive ability, and relatively severely affected by age. Older adults’ weak performance in this area may be the result of previously well-documented executive function impairments [53–56]. In contrast, AM detail is a measure of memory elaboration (remembering *how* an event unfolded) for which executive abilities appear to play a lesser role. These findings hint at the possibility that it is not memory, per se, that is impaired in healthy older adults, but rather executive processing that produces a deficit under circumstances when the executive requirement is high. Taken together, these findings suggest two possible routes for interventions aimed at supporting older adults’ memory: (a) the use of external memory aids to reduce the cognitive burden of search and construction processing, and (b) training executive function ability.

However, the exploratory analyses also suggested that different types of events were retrieved in each task. For example, in the Everyday Memory task, young adults used more words related to work and education, while older adults used more leisure words. This difference is unsurprising in that it reflects the common daily activities of young adults predominantly in full time work or education and older adult retirees. However, it may in part explain the absence of age effect on this task; in the main, work and education are repetitive activities, often dictated by schedules, involving daily commutes, a restricted range of locations and people, and, in many cases, long hours in front of a computer. In comparison, leisure activities may be more varied. If the events sampled by the young adults were more routine than those sampled by the older adults, this may have biased the task towards the superior performance of the older adults. Participants’ subjective ratings showed that events that happened less frequently were perceived to be more distinctive, and it was these events that were recalled in greater episodic detail at test (replicating our previous finding [13]).

The notion that routine events are less memorable seems obvious to the layperson, and is corroborated by studies from the developmental literature showing that even young children have difficulty recalling new episodic details about events that have been experienced multiple times [57, 58]. One way of understanding this is in terms of interference, whereby memories that share similar content or context compete with one another at retrieval, leading to poorer recall (e.g. [59]). Laboratory studies of interference have shown that interference increases as the amount of information in the system increases (e.g., when the number of learned items increases [60]). Thus, the more routine an event, the harder it should be to remember, even for young adults.

More generally, this interference effect has clear implications for older adults, who have a great many more years of accumulated lifetime experience than young adults. Since routine, repetition, and shared event contexts are abundant in everyday life, interference must surely increase with age. As a result, the task of recalling details unique to one specific event should be more difficult in older age due to the increased difficulty of resolving this interference.

Indeed, a parallel effect has already been demonstrated in two simulation studies of paired associate learning. In paired associate learning, participants are required to learn the association between pairs of words, each consisting of a cue and a response. At test, participants are presented with the cue and must produce the correct response. Typically, older adults perform at the same level as young adults when there is a pre-existing association between the words (e.g., up-down), but perform disproportionately poorly when there is no pre-existing association (e.g., up-car) [61]. Using word frequency data to simulate lifetime exposure to words, the two simulation studies showed that this pattern of age-related deficits can be explained by the increased information processing cost incurred by increased lifetime exposure in older adults [62, 63]. The authors argued that this is because the pairs are composed of highly familiar words that have been encountered frequently over the lifespan. Each exposure to a stimulus–in this case a word–not only strengthens its association with co-occurring stimuli, but also weakens its association with all other (non-co-occurring) stimuli. Thus, in paired associate learning, the more prior exposure one has had to the cue *in the absence of the response*, the harder it is subsequently to learn the novel association between the two. While this pattern is observed in both young and older adults, in older adults the effect is magnified by their greater total exposure across the lifespan.

In everyday life, very few events are entirely novel; even the most distinctive events usually involve the novel *combination* of some familiar features and some unfamiliar features–these could be themes, people, places, objects, goals, emotions, and so on. The interference account outlined above would predict that older adults, who have accumulated a great deal more experience of these features occurring independently, should have disproportionate difficulty remembering new associations between them. Conversely, older adults should find it relatively easier to remember pre-existing associations between features that commonly co-occur. Thus, we might expect to find that older adults exhibit a proportionately greater deficit in memory for novel events (involving novel combinations of familiar features), but relatively good memory for routines and repeated events (consisting of frequently co-occurring features). The results of the present study appear broadly consistent with this view, in that the smallest difference between groups was on the Everyday Memory task, which measured memory for relatively routine events. We did not, however, collect distinctiveness data for the memories that were retrieved in other tasks, and can only speculate that those that came readily to mind in response to the generic cues were likely to be those that were particularly distinctive.

The design of this study required us to make certain methodological decisions, and we now turn to a discussion of the advantages and disadvantages of our approach. Firstly, the five memory tasks were administered to all participants in the same sequence, in order of increasing cue specificity. The justification for this decision was to minimise the spillover effect of cues or memories in one task priming the retrieval of memories in a later task. Although this approach could not prevent a spillover effect, any such primed memories would be less likely to be appropriate in later tasks when the cues were more specific. For example, retrieval of a memory of a holiday in the word-cued AM task might prime retrieval of other memories of other holidays, but these would not be appropriate responses in the subsequent Everyday Memory task, which specified precisely which event was to be recalled.

Fixing the sequence of tasks for both groups also meant that, in our relatively small sample size, the difference in patterns between young and older groups could not be due to order effects. However, this approach did introduce task order as a potential confound, and this could have influenced the results in one of two ways. One possibility was that, if older adults fatigued more quickly than young adults, their performance on the later tests would be impaired. This did not appear to be the case, since the smallest between-group difference was on the final task, Everyday Memory. If older adults’ performance *was* impaired by fatigue, then the true age effect on this task would presumably be even smaller, or perhaps reversed, and this would not affect the conclusions of the study. Another possibility is that there was a “warming up” effect, whereby performance increased over the test session as participants became more relaxed, entered an optimal retrieval state (e.g. [64]) or were primed by earlier cues or responses. If this were the case, performance might be expected to improve across the test session, though it is not clear whether this should be expected to affect young and older adults at different rates, and it was not possible to look for trends within either group due to the different scoring protocols used in each task. There was no evidence for any task-order trend in the relative performance of young and older adults, which suggests that task order did not *cause* the pattern of results that were obtained, though we cannot rule out the possibility that the scores of either group were nudged by this factor.

Another consideration was that participants were only required to recall a single event in each of the Age 11–17 AM and Past Year AM tasks, and thus we cannot be confident that the retrieved memories were representative of their categories. Although this is true of both young and older adults’ responses, it may be more pertinent in assessing older adults’ performance, since to a greater extent than young adults, older adults appear to draw on a small pool of frequently repeated memories [65]. In very well-rehearsed memories, details that appear to be episodic may actually be remembered as a learned story (e.g. [66]) and may be functionally more similar to semantic memory details. The assessment of performance on this task may have been improved if the reliance on well-rehearsed memories could be reduced by requiring participants to recall a large number of events, however it is not clear what number would be appropriate, and in this instance we were constrained by the length of the test session.

Finally, previous studies have found that cue imageability [67] can affect AM specificity, as well as the likelihood of direct, as opposed to generative, retrieval [25, 68]; whether these factors interact with age is not yet known. In addition, there is some evidence that age effects are larger when cue words are neutral, compared to when cues are positively or negatively valenced [69]. It is therefore possible that a different set of cue words would produce different results.

## Conclusions

The findings here show that within a single sample of participants, different tasks used to measure AM vary in their sensitivity to ageing, and therefore cannot be considered interchangeable tests of AM ability. The reason for this varying sensitivity appears to be multifactorial. Memory specificity (or remembering *that* something happened) was influenced by executive function ability, and was particularly sensitive to age. In contrast, memory detail (remembering *how* something happened) was less sensitive to age, but was influenced by the retention interval at which events were recalled, as well as by event distinctiveness. Shorter retention interval and high distinctiveness were associated with recall of more episodic details. The extent to which these factors might interact with age remains unclear, and would be an interesting avenue for future work.

## Supporting information

S1 File Additional justification for, and details of, the analyses presented in the exploratory results section. (PDF)
