# Supplementary material for: Age effects in autobiographical memory depend on the measure
Source: PLoS One. 2021 Oct 29;16(10):e0259279. doi: 10.1371/journal.pone.0259279 (PMC8555790; doi:10.1371/journal.pone.0259279)
Supplement: S1 File — (PDF) [file pone.0259279.s001.pdf]

Supporting Information

Detailed justification and discussion of exploratory analyses: Episodic memory

Memory age

Perhaps the most obvious way in which the AM tasks differ from one another is the length of retention interval between the original experience and the memory test. Visual inspection of the plotted *z*-scores in *Figure 1* of the manuscript suggests that age effects in AM may increase with increasing retention interval. For example, between-group differences are smallest in Everyday Memory, when the retention interval is around 2 weeks, intermediate for Past Year AM, and larger for Age 11-17 AM, when the retention interval is several years or decades.

One possibility is that older adults forget more quickly than young adults. Indeed, there is some evidence for accelerated forgetting over relatively short periods (e.g., 1 hour) in older adults (Huppert & Kopelman, 1989; Mary, Schreiner, & Peigneux, 2013; Wheeler, 2000), though it is not clear whether this effect persists when initial encoding is controlled for (Fjell, Walhovd, Reinvang, et al., 2005), or whether any differences speed of forgetting would persist over much longer time frames (i.e., years).

Whether or not forgetting is faster in older adults, AM tests are frequently biased towards superior recall in young adults because the retention intervals are shorter for younger adults. This may be the case either because age-at-encoding is matched, and thus retention interval must necessarily be longer for older adults (as in the case of Age 11-17 AM), or because the time period is unconstrained (as in the case of Word-Cued AM), in which case older adults tend to recall older memories than young adults (Hyland & Ackerman, 1988; Sperbeck, Whitbourne, & Hoyer, 1986). When both participant age and memory age covary, it is difficult to disentangle the effects of each, since both are associated with memory “semanticisation” (reduced episodic detail with or without an increase in semantic detail, e.g., Cabeza & St. Jacques, 2007; Conway, 2009; Moscovitch, Nadel, Winocur, Gilboa, & Rosenbaum, 2006; Nadel & Moscovitch, 1997; Smith & Graesser, 1981). Thus, in our data, what appear to be effects of age might in fact be effects of retention interval.

To investigate this, first each memory from the Word-Cued AM task was rated according to its age (1 = 0-5 years; 2 = 6-10 years; 3 = 11-20 years; 4 = more than 20 years). Memories were rated retrospectively by the experimenter, because participants were not originally asked to date the memories, but in most cases they voluntarily reported information about the time period (e.g., *when I was 21*; *last year; in 2006*). A number of responses did not contain sufficient information to be

assigned to a time period, and some responses could not be dated because they were repeated or extended over a period that spanned two or more age bins, because they were too vague, or because the participant had failed to recall anything at all. There were eight such responses across seven participants in the younger group and 22 such responses across 12 participants in the older group. These responses were excluded before calculating the binned mean retention interval for each participant across the remainder of the memories recalled for this task. In line with previous findings, the mean retention interval for younger adults (M=1.32, SD=.35) was significantly shorter than for older adults (M=2.24, SD=1.01; $t(38)=3.85$, $p<.0005$). *Fig. S1* shows the age of the memories recalled by both groups. There were not enough data to compare memory specificity at each interval, therefore the specificity of younger and older adults' memories was compared for the most recent period only (0-5 years). Three older participants were excluded from this analysis because they did not recall any memories fewer than five years old. For the remaining sample (n=37), the mean specificity rating was calculated for all eligible memories to give a single score per participant. Group comparison revealed that young adults' memories (M=3.57, SD=.50) were still more specific than older adults' memories (M=3.03, SD=.47; $t(35)=3.36$, p=.002), thus increased retention interval could not explain older adults' poorer performance on this task.

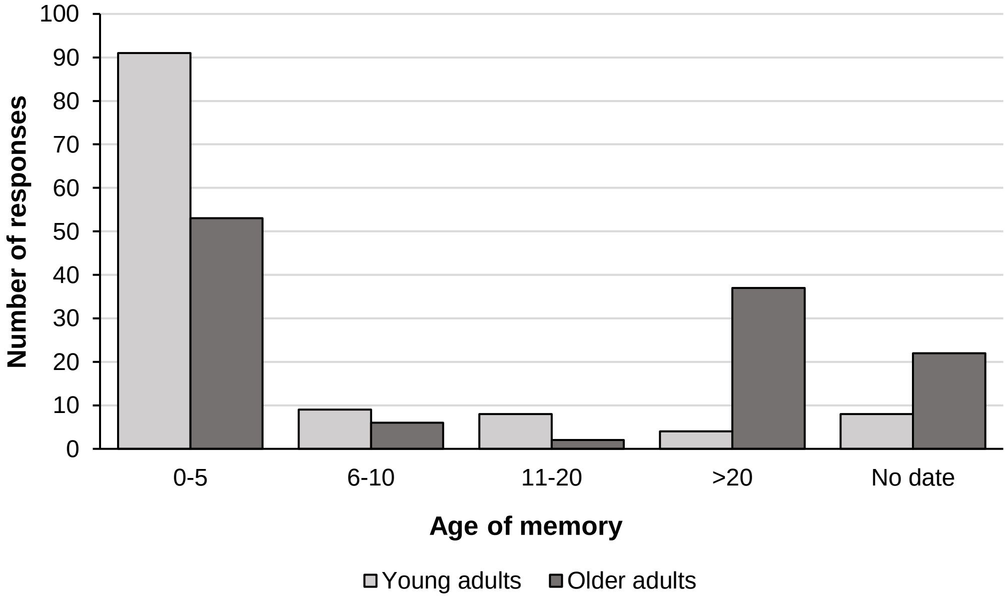

Fig. S1. Approximate age of memories (in years) recalled by older and younger adults on the word-cued AM task

Supporting Information

We also investigated the effect of retention interval by comparing Past Year AM and the Age 11-17 AM in a 2 (age group) x 2 (task) ANOVA. Here, the task instructions and coding protocol were identical, but retention interval was (a) much longer for Age 11-17 AM, and (b) matched in Past Year AM but mismatched in Age 11-17 AM. As predicted by accounts of memory semanticisation, there was a main effect of retention interval ($F(1,38)=7.71$, $p=.008$, $\eta^2=.17$, see *Fig. 3*) in which fewer episodic details were recalled for the older memories. However, the interaction between age group and task was not significant ($F(1,38)=.49$, $p=.49$, $\eta^2=.01$).

The lack of interaction may be due to low statistical power, since visual inspection of *Fig. S2* suggests that the between-group difference in Age 11-17 AM was approximately twice as large as the between-group difference in Past Year AM. However, in Age 11-17 AM the retention interval for older adults (~55 years) was around 5 times longer than the young adults' interval (~10 years); if the effect of retention interval is assumed to be linear, this would suggest that older adults were performing surprisingly well. To illustrate, if it is assumed that these two tasks represent two levels of a single experimental condition "retention interval", we see that young adults' memories lost, on average, around 8 details over 10 years. Assuming a linear forgetting function, and a similar rate of forgetting for older adults, extrapolation from the starting point of 29 details in Past Year AM suggests that older adults should not recall any details after 40 years.

Supporting Information

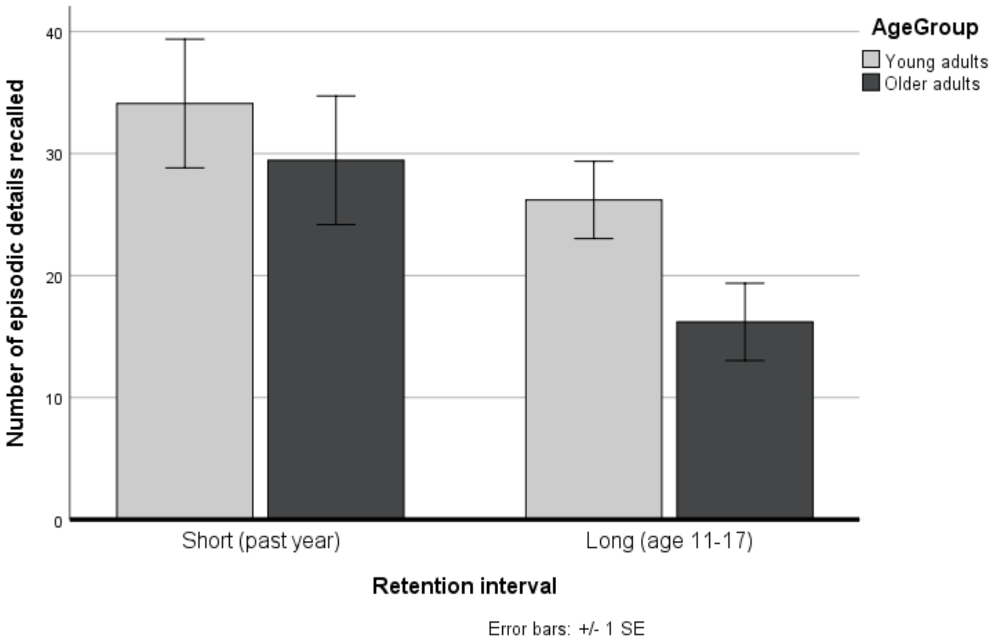

Fig S2. Number of episodic details recalled by older and younger adults in two comparable AM tasks (Past Year AM, Age 11-17 AM), which differed in retention interval.

However, previous analyses of the distribution of AMs across the lifespan have shown that they follow a power function, that is, the distribution is linear on a logarithmic scale, with a base of 2 (Rubin & Schulkind, 1997). If we assume that forgetting follows the same power function (Wixted & Ebbesen, 1997), then scores on the two tasks can be converted to base 2 logarithms, plotted as linear functions, and the rate of forgetting in young adults and older adults can be compared (see *Fig. 4*). The forgetting slope for older adults ($y = -0.12x + 4.88$) is steeper than the forgetting slope for young adults ($y = -0.09x + 5.09$), thus older adults' performance on the age 11-17 AM task was poorer than expected, relative to their performance on the past year AM task (*Fig. S3*).

Supporting Information

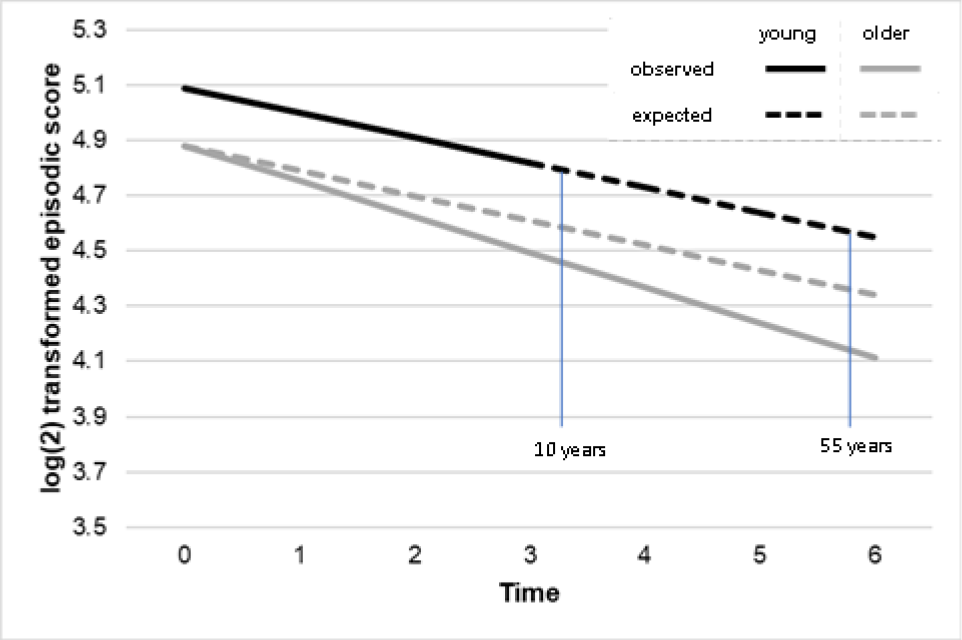

Fig. S3. Forgetting over time, based on scores in Past Year AM and Age 11-17 AM. Forgetting is assumed to follow a power function, therefore scores have been log-transformed and plotted linearly. This facilitates comparison of young and older adults, for whom retention intervals differed substantially in Age 11-17 AM. Young adults are represented by the black line, and older adults are represented by the grey lines. Solid lines represent observed data, while dotted lines represent predicted scores based on the observed score for Past Year AM (time = 0) and the rate of forgetting for young adults. Thus, the dotted black line shows the predicted score for young adults over a much longer retention interval than was observed (time = 3.25-6), and the dotted grey line shows the predicted performance for older adults assuming the rate of forgetting was equal to the rate of forgetting for young adults. The solid grey line shows that older adults' performance on the Age 11-17 AM task (time = 5.72) is poorer than would be expected if retention interval was the only predictor of performance, and the rate of forgetting was equivalent to the rate of forgetting for young adults.

Supporting Information

A limitation of requiring participants only to recall one event from each time period, however, is that the sampled memory may not be representative of recall for that period. The participant is permitted a great deal of flexibility in interpreting the cue, and may default to the most readily accessible memories, which are probably those that are well-rehearsed. This sampling technique may therefore produce memories that appear to contain many episodic details, but these are not truly episodic in the sense of being accompanied by the experience of mental time travel/subjective reliving. This kind of false episodic memory was described in an amnesic patient whose episodic memory was impaired, but who was still able to recite the details of life events, which he appeared to have learned as though they were semantic stories (Patient SS; Cermak & O'Connor, 1983). Thus, for very well-rehearsed memories it is difficult to establish whether the retrieved event details are truly episodic. While this is likely to be true of both young and older adults, there is some evidence to suggest that, to a greater extent than young adults, older adults tend to draw on a small pool of frequently repeated memories (Cohen & Faulkner, 1988). Moreover, it seems likely that memories retrieved after longer retention intervals may have been subject to more rehearsal, in total, than more recent memories. Consequently, even when instructions, materials, and scoring protocols are held constant, it is not clear that tasks comparing memories across different retention intervals are measuring the same ability (i.e., if recent memories are more likely to involve "true" episodic memory, and remote memories are more likely to involve "semanticised" learned stories).

The representativeness issue could be addressed by asking participants to describe a large number of memories for each retrieval cue, and discounting those produced earlier in the output, which are likely to be the most well-rehearsed. However, the number of memories that would have to be discounted is unknown, and may well vary between participants. Another possibility might be to constrict the retrieval cue in some way, such that the participant has less flexibility in interpreting the cue, which should in turn reduce reliance on readily available learned stories. However, this runs the risk of failing to elicit a memory at all. It is difficult to balance these issues experimentally, especially in ageing research, and future work may benefit from a radically different approach to cueing AM.

Executive function

Another non-memory factor that could contribute to differences in task performance is executive function. For example, different types of retrieval cue may vary in the demand they place on effortful

generative retrieval processing – an executive “working-with-memory” faculty (Moscovitch, 1992) that appears to be particularly difficult when executive capacity is compromised (Williams et al., 2006). Specific cues should, theoretically, place less demand on executive processing than general cues, because specific cues more often lead to direct retrieval, without the need for cue elaboration and iterative search for an appropriate memory to describe (Conway & Pleydell-Pearce, 2000). Similarly, previous work has shown that personalised cues lead to direct retrieval more frequently than generic cues, which require generative processing (Uzer & Brown, 2017). Tasks that require a greater amount of generative processing may be more sensitive to ageing, given evidence of age-related declines in executive functions (Berna, Schönknecht, Seidl, Toro & Schröder, 2012; Clarys, Bugaiska, Tapia, & Baudouin, 2009; Fisk & Sharp, 2004; Lee, Crawford, Henry, et al., 2012).

The selection of tasks presented in the manuscript varied in the specificity of cues used to elicit memories. Everyday Memory cues were both specific and personalised (having been generated by the participants themselves), whereas cues for Past Year AM, Age 11-17 AM, and Word-Cued AM were generic and non-specific. We therefore investigated whether the tasks most sensitive to age were those with the highest executive demand.

Supporting Information

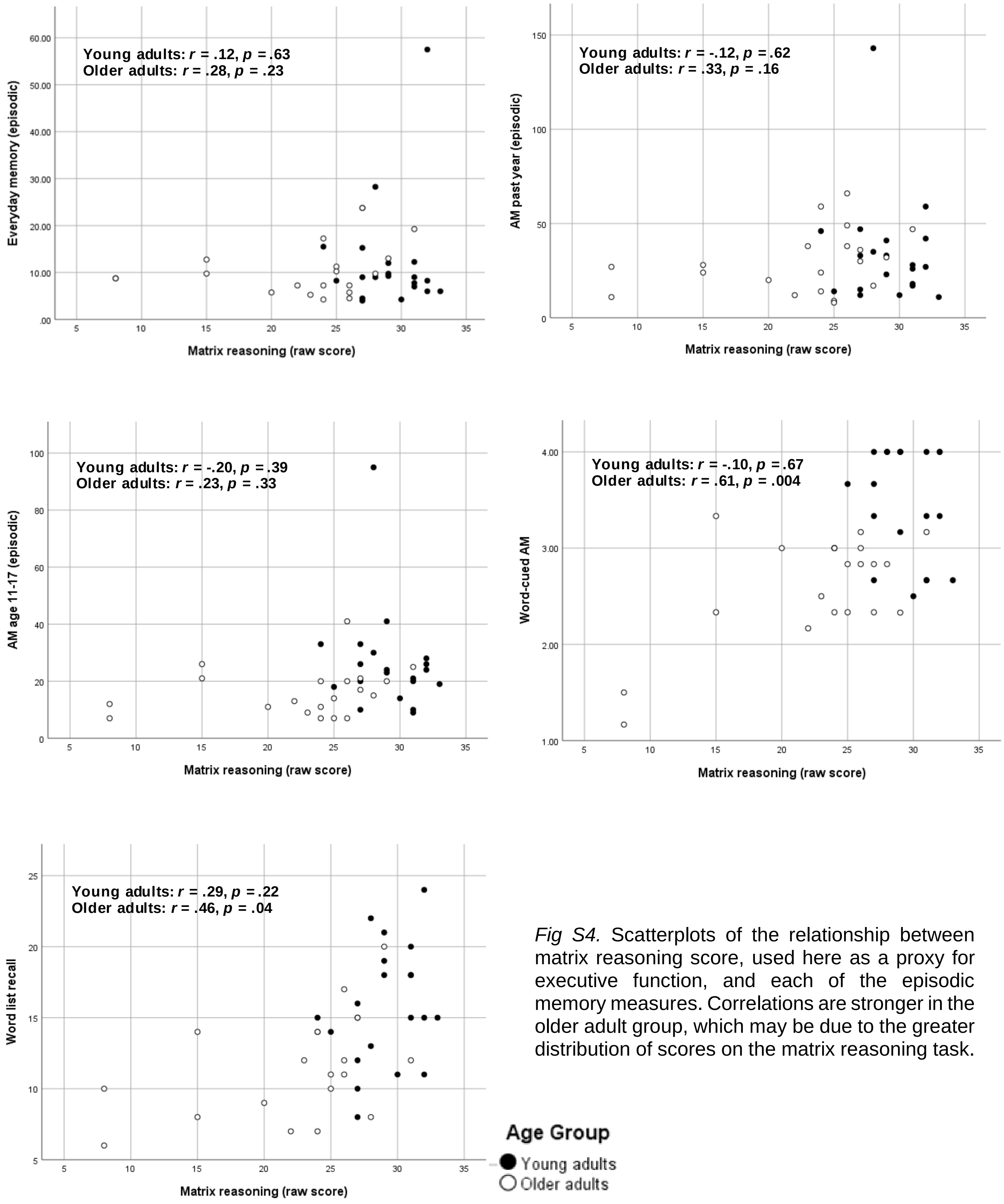

Fig S4. Scatterplots of the relationship between matrix reasoning score, used here as a proxy for executive function, and each of the episodic memory measures. Correlations are stronger in the older adult group, which may be due to the greater distribution of scores on the matrix reasoning task.

Supporting Information

Although no direct measures of executive function were administered, the matrix reasoning subtest of the WASI involves similar abilities (working memory, inhibition, updating) and is moderately to strongly correlated with executive function (Friedman, Miyake, Corley, Young, DeFries, & Hewitt, 2006; Roca, Manes, Chade, et al., 2012). Using matrix reasoning as a proxy for executive function, we therefore calculated correlations between raw (unstandardized) matrix reasoning scores and each episodic measure, and found that the strength of the relationship varied across tasks. Matrix reasoning was clearly correlated with Word-Cued AM (r=.59, p<.0005) and Word List Recall (r=.53, p<.0005), but was not significantly correlated with the number of episodic details recalled in Everyday Memory (r=.17, p=.30), Past Year AM (r=.15, p=.36), or Age 11-17 AM (r=.21, p=.19).

Since matrix reasoning scores were significantly higher for younger, relative to older, adults (t(38)=4.23, p<.0005), the pattern of correlations between matrix reasoning and memory measures could be caused by this underlying age effect. We therefore calculated the correlations between matrix reasoning performance and each episodic measure separately for each age group. Scatterplots with correlation statistics are presented in *Fig. S4*, and show that the pattern of correlations described above was evident in the older adult group, but in the young adult group the relationship was less clear, and three of the five coefficients were negative. The pattern of correlations did not suggest that matrix reasoning ability alone could account for the variable age effect in AM, since Everyday Memory, Past Year AM, and Age 11-17 AM each had a similar relationship with matrix reasoning, despite varying age effects. However, the correlations do appear to show that tasks can be dissociated by their relationship to executive function, that is, some tasks (Word-Cued AM, Word List Recall) are more executively demanding than others (Everyday Memory, Past Year AM, Age 11-17 AM). Older adults appear to perform poorly on tasks with a high executive demand, while their performance on tasks with lower executive demand is mixed, and may be more dependent on other factors.

Depression

Another factor that has been shown to covary with both executive function and episodic memory retrieval is depression. Although we did not initially plan to look at depression as a contributing factor, we administered the Geriatric Depression Scale (GDS) to all participants as a way of comparing the young and older groups, in addition to comparing IQ and education. As reported in the main text of

the manuscript, GDS scores did not differ between the two groups, but GDS was negatively correlated with Matrix Reasoning scores, and the association was stronger for older adults than younger adults. In the manuscript we did not discuss depression further because, across the whole sample, there was no significant correlation between GDS and any of the memory measures. However, six participants scored above the GDS cut-off of 9, indicative of symptoms consistent with clinical depression. Four of these participants were in the young adult group (scores = 9, 9, 10, 12), and two were in the older adult group (scores = 15, 23). In these six participants, GDS score was strongly negatively correlated with Word-Cued AM and Word List Recall. *Table S1* shows the correlations between GDS and memory, in participants who scored above and below the cut-off. It is difficult to interpret these correlations since the participants with the highest GDS scores were also in the older adult group. However, in an attempt to determine whether depressive symptoms affected the findings, we re-ran the 2 (age group) x 5 (episodic memory measure) ANOVA reported in the manuscript as an ANCOVA, with GDS as the covariate (for this analysis n=39, due to missing GDS score for one older adult). Results showed an interaction between age and memory measure ($F(4,144)=3.40$, $p=.01$, $\eta_p^2=.09$) that was of similar magnitude to the interaction reported in the manuscript, thus adding depression as a covariate did not alter the findings.

Table S1
Correlations between GDS and memory

	Depressed (n=6)	Not depressed (n=34)
Word List Recall	-.60	-.06
Word-Cued AM	-.74	-.17
Age 11-17 AM	-.11	-.06
Past Year AM	-.08	-.10
Everyday Memory	-.18	-.11

Event type

The final between-task difference we investigated was the type of event that was described in each. Clearly, the to-be-remembered stimuli in a word-list-learning task differ substantially from the to-be-remembered events in a more naturalistic memory task. However, even within naturalistic memory, events can vary in their importance to one’s life story (significant vs. everyday events), their

distinctiveness (how much they stand out relative to other events), their emotional intensity, the extent to which they overlap with other similar events (repeated vs. unique events), the extent to which they have been rehearsed, and so on. Many of these factors are likely to overlap, and result in events that vary in their *memorability*, independently of the *memory ability* of the remember. The memorability of different types of events has received surprisingly little attention in AM, especially in relation to ageing.

In the main text of the manuscript, we showed in the Everyday Memory task that events that took place more frequently were less distinctive, and were remembered in less detail (see further details of this analysis below); this was true for both young and older adults. There are several reasons to assume that the variability in the memorability of events would also be observed between tasks. For example, tasks vary in terms of their specification of a time period from which to sample AMs. When the task is to recall events from a short period, such as the past week, the type of events available for recall are likely to be substantially different from the type of events available for recall from a longer period, such as the past year. Events from the past week are more likely to include mundane everyday activities such as going to work, the daily commute, the weekly shop, and so on; these are things that happen regularly. In contrast, events from the past year are more likely to include distinctive events such as parties, holidays, and so on, which take place less frequently and so are less likely to be captured during a shorter sampling period. Presumably if the time period is further extended to a decade, or perhaps even a lifetime, the distinctiveness of the events also increases such that the chance of sampling once-in-a-lifetime experiences (e.g., a wedding, the birth of a first child) increases. The length of the sampling period is therefore likely to affect the memorability of sampled events.

In addition, the remoteness of the sampling period is also likely to affect event memorability. Most of the mundane activities that take place in any given week would simply not be readily accessible at a retention interval of longer than a few weeks. It is difficult to recall what one had for lunch on the second Tuesday of last month (unless one has the same thing for lunch every day, or it happened to be a special occasion). In contrast, more distinctive activities such as holidays and weddings are easier to retrieve after a long interval precisely because they happen less frequently.

Supporting Information

Thus, the time period from which memories are sampled is an important consideration for participants of any age.

However, additional complications arise when comparing young and older adults because matching the retention interval across age groups causes a bias in age-at-encoding, and matching age-at-encoding causes a bias in retention interval. When age-at-encoding is matched, young and older participants tend to describe similar types of AM, but older adults' do so at much longer retention intervals. For example, in one study, both groups described memories about education from the first two decades of life, and memories about births, marriages, and deaths from the third decade (Cohen & Faulkner, 1988). When there is no restriction on age-at-encoding, there are significant differences in the topics covered by young and older adults – at least in the 1980s, older participants were more likely to describe memories of war and illness, whereas younger participants were more likely to describe memories of love, sex, education, and hobbies (Cohen & Faulkner, 1988). Differences in the topics of AMs do not necessarily translate to differences in the memorability of the events, but it is possible that certain topics, such as war, elicit particularly distinctive memories. This only serves to highlight that relatively little is known about the qualitative content of retrieved AMs, and whether this bears any relation to the quantity of information that is recalled.

Using data from the present study, we therefore explored the content of the retrieved events using textual analysis software (Linguistic Analysis and Word Count; LIWC; Pennebaker, Booth, Boyd, & Francis, 2015) to see whether any distinct patterns emerged within and between tasks. This analysis follows the event frequency and distinctiveness analysis below.

Further details of frequency/distinctiveness analysis. In the Everyday Memory task, since participants recalled four separate memories, there were four scores per participant for each variable and the data were hierarchical, which compromised statistical independence (Wright, 1998). To account for person-level variance within these data, we first calculated the within-subject correlations between each pair of variables, across the four ratings per participant. It was not possible to calculate a correlation coefficient for distinctiveness in one older participant because the variable was continuous (i.e., a distinctiveness rating of 1 was given to all events).

We then calculated the mean correlation for each pair of variables across the whole sample, and conducted one-sample *t*-tests, corrected for multiple comparisons (α =.005) to determine whether

the correlation coefficients were statistically different from zero. The mean correlations are presented in *Table S2,* first for the full sample and then for young and older adults separately.

The relationships between frequency, distinctiveness, and episodic detail replicated those found in our previous study (Mair et al., 2017). In the full sample there was a negative correlation between the number of episodic details and event frequency ratings ($t(39)=-6.10$, $p<.0005$), and between event frequency and event distinctiveness ($t(38)=-10.80$, $p<.0005$), and there was a positive correlation between episodic details and event distinctiveness ($t(38)=5.45$, $p<.0005$). The same pattern of correlations was observed in young and older adults independently, and suggests that events are more memorable if they take place less often, and if they stand out more from one's usual activities at the time they are experienced. This finding should be uncontroversial, since it is entirely consistent with lay intuitions about memory, but it serves to highlight the fact that not all events are equally memorable, and as well as individual differences in memory ability, some of what the different AM tasks measure may be individual differences in the memorability of events.

Table S2
Mean within-subject correlations between event characteristics and episodic and semantic recall in the everyday memory task

		Distinctiveness	Episodic detail
Overall	Frequency	-.66	-.48
	Distinctiveness		.42
Young adults	Frequency	-.63	-.51
	Distinctiveness		.40
Older adults	Frequency	-.69	-.46
	Distinctiveness		.44

Text analysis. Since prospective frequency/distinctiveness ratings were not available for the other AM tasks, we explored some of the features of the memories reported in each task using LIWC. LIWC is a programme that reads raw texts and categorises the words in relation to an inbuilt dictionary of psychological themes, narrative styles, and parts of speech, which have been shown to correlate with various psychological constructs (Tausczik & Pennebaker, 2010). To our knowledge

there is no published work in which LIWC has been used to examine AM content, and the measured constructs are not memory-specific. However, it provides a replicable way of examining memory narratives in more depth than a detail count. Of course narrative style is idiosyncratic, but the within-subjects design means that each participant acts as their own control, and therefore differences between tasks could be informative.

Fig. S5 shows a selection of LIWC output categories plotted by age group and task, and suggests that memories recalled in each of the tasks differed in some interesting ways. However, in the absence of any theoretical or practical framework for this analysis these differences should be interpreted with caution. We did not perform inferential analyses because of the large number of categories; therefore the plots below are used only to describe the current data and propose some very tentative hypotheses for future investigations. The LIWC categories plotted in *Fig. S5* are only a subset of the categories in the default LIWC output but the full LIWC output is available on the OSF project page (https://osf.io/9wxyz/). A list of brief descriptions of the memories recalled by young and older adults in each task is available on the same OSF project page and can be used alongside the interpretations offered below.

The patterns of word usage were broadly the same for young and older adults; both groups' memories were similarly emotional, covered similar themes, and had similar temporal focus. The patterns varied somewhat across tasks, and the task differences appeared to be largely comparable for both groups.

In both young and older adults, Age 11-17 AMs had a relatively high proportion of words related to the theme of "work" (including education words, e.g., job, class, exam, pen, write, pay), and tended to use first person singular pronouns (I, me, mine), whereas Past Year AMs had a relatively high proportion of words relating to the theme of "leisure" (e.g., museum, cinema, drink, fun, shop, weekend), and used a greater proportion of first-person plural pronouns (we, us, our). Age 11-17 AMs therefore seemed to describe events experienced as an individual and, given the sampled time period, probably described days at school or a first job. On the other hand, memories from the past year seemed to describe social events experienced alongside other people (as indicated by the plural pronouns). Both Age 11-17 AMs and Past Year AMs contained a relatively high proportion of past-tense words ("past focus"), and a relatively low proportion of present-tense words ("present-

focus”). This perhaps suggests that these memories described closed chapters that had relatively little overlap with the participants’ present situation.

Word-Cued AMs contained a relatively low proportion of social words (e.g., mate, talk, they, child) but were high in positive emotion words (e.g., accept, like, love, special, hope, happy) – unsurprising given that three of the six cue words were positively valenced. These memories were also high in first-person singular pronouns and contained a relatively high proportion of both past-tense and present-tense words. This pattern suggests that the sampled events described important or salient *individual* experiences (as indicated by the combination of first-person singular pronouns and few social words) that perhaps had some ongoing relevance to the participant’s current state, or prompted reflection and interpretation of the events in the present (as indicated by the higher proportion of present-tense words).

In contrast, Everyday Memories were comparatively *low* in past-focus, but were high in present-focus, likely because in this task participants frequently described events that were part of current or ongoing routines (e.g., grocery shopping) and involved relationships that were still relevant at the time of retrieval (e.g., drinks with friends). They contained a relatively high proportion of social words but were low in emotion and made more references to money (e.g., buy, sale, shop, expensive) than memories recalled in the other tasks, suggesting that although these events frequently referred to other people, they were relatively mundane. For young adults, these memories contained a high proportion of words relating to work, while for older adults there were more words relating to leisure, reflecting the different day-to-day engagements of the young participants in work or full-time study, and the over 65s who had retired.

The different AM tasks therefore appeared to elicit memories of different types of events; these varied in terms of emotion, sociality (individual/social), theme (work/leisure), and perhaps their relevance to ongoing activities (i.e., whether or not the event represented a “closed chapter”). The different tasks also appeared to highlight cohort differences in, for example, everyday experiences. Some of these differences could affect the memorability of the retrieved events.

Supporting Information

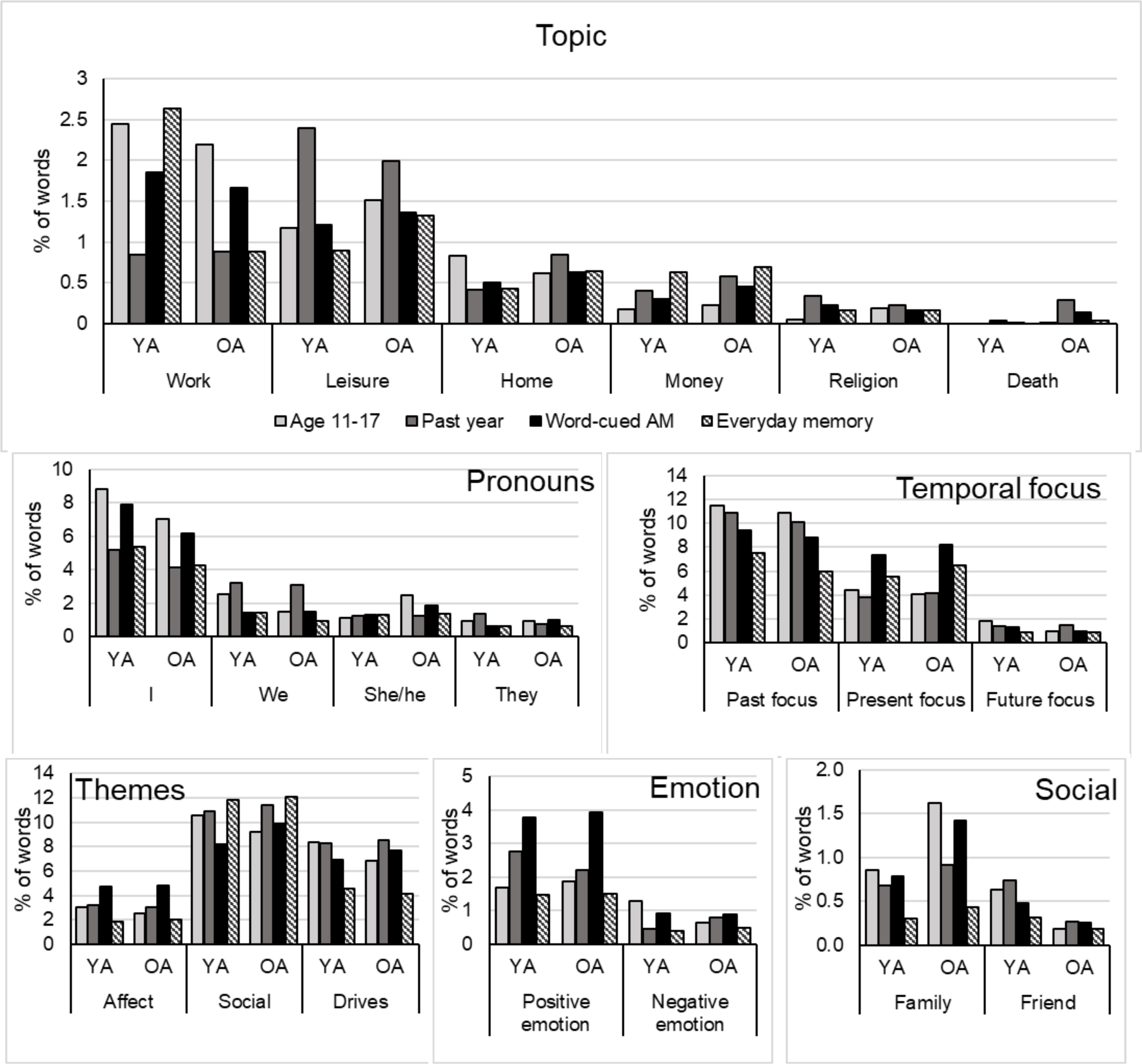

Fig. S5. Selected characteristics of memories reported by young and older adults in each narrative memory task, analysed using Linguistic Inquiry and Word Count software (LIWC; Pennebaker et al., 2015). Note. YA = young adults; OA = older adults.

We discussed in the main manuscript text the possibility that older adults performed relatively well in the Everyday Memory task because in this task event sampling was biased towards more distinctive events in the older group. Although this hypothesis did not appear to be supported by the data – the self-rated distinctiveness of the sampled events did not differ between groups – it seems

likely that each group's ratings were benchmarked against other recent experiences, rather than experiences across the lifespan. As a result, the threshold for considering any given event distinctive likely differed between individuals, meaning only the relative ratings given by each participant would be informative.

Further analyses of semantic recall

Increased semantic AM in older adults is a robust finding, but the relationship between episodic and semantic AM is not clear. One hypothesis is that semantic AM in older adults serves to compensate for a deficit in episodic AM, since the increase in semantic details is often observed in the context of a reduction in episodic details (e.g., Levine et al., 2002). One recent study used multilevel analysis to show that, at the event level, the relationship between episodic and semantic recall was negative in three out of five studied datasets, particularly for older adults, and this was interpreted as evidence that the external details were used to compensate for weaker episodic recall (Devitt, Addis, & Schacter, 2017).

Since we measured both episodic and semantic recall on three tasks in the present study (Everyday Memory, Past Year AM, and Age 11-17 AM), we explored the relationship between episodic and semantic details in each. There was a strong positive correlation (r=.61, p<.0005) in Past Year AM, a weaker positive correlation in Everyday Memory that was not significant (r=.17, p=.30), and no relationship in Age 11-17 AM (r=-.05, p=.76). Although semantic recall did not appear to compensate for poor episodic recall in any task, these simple correlations describe variance at the participant level. The positive associations could therefore be the result of interindividual differences in verbosity, especially since semantic recall was correlated across tasks (see *Table S3*).

Table S3
Correlations between tasks in semantic recall

	Past Year AM	Age 11-17 AM
Everyday Memory	.43**	.33*
Past Year AM		.45**

*p<.05; **p<.01

To examine the association between episodic and semantic recall at the event level, we carried out hierarchical correlation analyses of the two types of detail in Everyday Memory, since in that task there were four observations per participant. Episodic and semantic details were correlated within-participants, across the four observations, and the mean of the coefficients was calculated to give the average correlation across the

sample. The results are presented in Table S4, and show that, overall, there was no relationship between episodic and semantic recall. There was a small negative coefficient in the older adult group, but the large standard deviation indicated a great deal of variability between participants. We calculated further hierarchical correlations between semantic recall and event frequency and distinctiveness (see *Table S4*), to explore whether semantic details might be more prevalent in high-frequency, low-distinctiveness events, but did not find evidence for this. Although correlations between semantic recall and event frequency were, on average, positive, there was a great deal of variability between participants.

Table S4
Mean within-subjects correlation between semantic recall and episodic recall, event frequency, and event distinctiveness in the everyday memory task (SD in parenthesis)

Correlated variables	Full sample	Young adults	Older adults
Semantic details & episodic details	-.08 (.60)	.01 (.59)	-.17 (.61)
Semantic details & event frequency	.14 (.58)	.17 (.56)	.11 (.62)
Semantic details & event distinctiveness	.08 (.57)	.01 (.54)	.15 (.60)

One difficulty in interpreting semantic AM is that it is a heterogeneous category of details. Whereas episodic and semantic memory have classically been understood to be completely separate, current theories of episodic and semantic *autobiographical* memory argue that they rely on the same underlying knowledge base (Conway, Justice, & D'Argembeau, 2019; Rubin & Umanath, 2015). Accordingly, episodic and semantic AM have been argued to represent opposite ends of a continuum of abstraction, such that information can be represented in memory in a more episodic-like or semantic-like way (e.g., see Renoult et al., 2012). As it has been previously defined, episodic AM – pertaining to a unique event lasting less than a day – occupies one extreme of this continuum. In contrast, semantic AM incorporates a much broader spectrum of details, ranging from "experience-near" repeated events (e.g., *childhood Christmases*) and temporally extended events (*e.g., holiday in Italy*), to "experience-far" autobiographical knowledge (e.g., *John is my brother*) and general semantic knowledge (e.g., *Paris is the capital of France*). There is evidence that these different subcategories of semantic AM involve different brain areas (Grilli & Verfaellie, 2014, 2016)

and can be distinguished electrophysiologically (Renoult, Tanguay, Beaudry, et al., 2016). Recently, a reanalysis of existing data also found that people with Alzheimer's disease and semantic dementia could be dissociated by the profile of semantic AM subcategories within their memory narratives (Strikwerda-Brown et al., 2019). These findings suggest that analysing the subcategories of semantic AM may be more informative than considering the category as a whole, and could shed light on the reason for increased semantic AM in older adults.

References for supporting information

Berna, F., Schönknecht, P., Seidl, U., Toro, P., & Schröder, J. (2012). Episodic autobiographical memory in normal aging and mild cognitive impairment: A population-based study. *Psychiatry Research, 200,* 807-812.

Cabeza, R. & St. Jacques, P. (2007). Functional neuroimaging of autobiographical memory. *Trends in Cognitive Sciences, 11 (5),* 219-227.

Cermak, L. S. & O'Connor, M. (1983). The anterograde and retrograde retrieval ability of a patient with amnesia due to encephalitis. *Neuropsychologia, 21 (3),* 213-234.

Clarys, D., Bugaiska, A., Tapia, G. & Baudouin, A. (2009). Ageing, remembering, and executive function. *Memory, 17 (2),* 158-168.

Cohen, G. & Faulkner, D. (1988). *Life-span changes in autobiographical memory.* In M. M. Gruneberg, P. E. Morris & R. N. Sykes (Eds.), Practical Aspects of Memory: Current Research and Issues, Vol. 1: Memory in Everday Life, pp. 277-282. Oxford, UK: John Wiley & Sons.

Conway, M. A. & Pleydell-Pearce, C. W. (2000). Construction of autobiographical memories in the Self-Memory System. *Psychological Review, 107 (2),* 261-288.

Conway, M. A. (2009). Episodic memories. *Neuropsychologia, 47,* 2305-2313.

Conway, M. A., Justice, L. V., & D'Argembeau, A. (2019). The self-memory system revisited: Past, present, and future. In J. Mace (Ed.), *Structure and Function of Autobiographical Memory*. Oxford University Press, pp. 28-51.

Devitt, A. L., Addis, D. R., & Schacter, D. L. (2017). Episodic and semantic content of memory and imagination: A multilevel analysis. *Memory & Cognition, 45,* 1078-1094.

Fisk, J. E. & Sharp, C. A. (2004). Age-related impairment in executive functioning: Updating, inhibition, shifting, and access. *Journal of Clinical and Experimental Neuropsychology, 26 (7),* 874-890.

Fjell, A. M., Walhovd, K. B., Reinvang, I., Lundervold, A., Dale, A. M., Quinn, B. T., Makris, N., & Fischl, B. (2005). Age does not increase the rate of forgetting over weeks--Neuroanatomical volumes and visual memory across the adult life-span. *Journal of the International Neuropsychological Society, 11,* 2-15.

Friedman, N., Miyake, A., Corley, R. P., Young, S. E., DeFries, J. C., & Hewitt, J. K. (2006). Not all executive functions are related to intelligence. *Psychological Science, 17 (2),* 172-179.

Grilli, M. D. & Verfaellie, M. (2014). Personal semantic memory: Insights from neuropsychological research on amnesia. *Neuropsychologia, 61,* 56-64.

Grilli, M. D. & Verfaellie, M. (2016). Experience-near but not experience-far autobiographical facts depend on the medial temporal lobe for retrieval: Evidence from amnesia. *Neuropsychologia, 81*, 180-185.

Supporting Information

Huppert, F. A. & Kopelman, M. D. (1989). Rates of forgetting in normal ageing: A comparison with dementia. *Neuropsychologia, 27 (6),* 849-860.

Hyland, D. T. & Ackerman, A. M. (1988). Reminiscence and autobiographical memory in the study of the personal past. *Journal of Gerontology: Psychological Sciences, 43 (2),* 35-39.

Lee, T., Crawford, J. D., Henry, J. D., Trollor, J. N., Kochan, N. A., Wright, M. J., Ames, D., Brodaty, H. & Sachdev, P. S. (2012). Mediating effects of processing speed and executive functions in age-related differences in episodic memory performance: A cross-validation study. *Neuropsychology, 26 (6),* 776-784.

Levine, B., Svoboda, E., Hay, J. F., Winocur, G. & Moscovitch, M. (2002). Aging and autobiographical memory: Dissociating episodic from semantic retrieval. *Psychology and Aging, 17 (4),* 677-689.

Mair, A., Poirier, M., & Conway, M. A. (2017). Supporting older and younger adults' memory for recent everyday events: a prospective sampling study using SenseCam. *Consciousness & Cognition, 49,* 190-202*.*

Mary, A., Schreiner, S., & Peigneux, P. (2013). Accelerated long-term forgetting in aging and intra-sleep awakenings. *Frontiers in Psychology, 4:* 750.

Moscovitch, M. (1992). Memory and working-with-memory: A component process model based on modules and central systems. *Journal of Cognitive Neuroscience, 4 (3),* 257-267.

Moscovitch, M., Nadel, L., Winocur, G., Gilboa, A., & Rosenbaum, R. S. (2006). The cognitive neuroscience of remote episodic, semantic and spatial memory. *Current Opinion in Neurobiology, 16,* 179-190.

Nadel, L., & Moscovitch, M. (1997). Memory consolidation, retrograde amnesia and the hippocampal complex. *Current Opinion in Neurobiology, 7,* 217-227.

Pennebaker, J. W., Boyd, R. L., Jordan, K., & Blackburn, K. (2015). *The development and psychometric properties of LIWC2015.* Austin, TX: University of Texas at Austin.

Renoult, L., Tanguay, A., Beaudry, M., Tavakoli, P., Rabipour, S., Campbell, K., Moscovitch, M., Levine, B., & Davidson, P. S. R. (2015). Personal semantics: Is it distinct from episodic and semantic memory? An electrophysiological study of memory for autobiographical facts and repeated events in honor of Shlomo Bentin. *Neuropsychologia, 83,* 242-256.

Roca, M., Manes, F., Chade, A., Gleichgerrcht, E., Gershanik, O., Arévalo, G. G., Torralva, T., & Duncan, J. (2012). *Psychological Medicine, 42,* 2445-2452.

Rubin, D. C., & Schulkind, M. D. (1997). Distribution of important and word-cued autobiographical memories in 20-, 30-, and 70-year old adults. *Psychology and Aging, 12 (3),* 524-535.

Rubin, D. C., & Umanath, S. (2015). Event memory: A theory of memory for laboratory, autobiographical, and fictional events. *Psychological Review, 122 (1),* 1-23.

Smith, D. A. & Graesser, A. C. (1981). Memory for actions in scripted activities as a function of typicality, retention interval, and retrieval task. *Memory & Cognition, 9 (6),* 550-559.

Sperbeck, D. J., Whitbourne, S. K. & Hoyer, W. J. (1986). Age and openness to experience in autobiographical memory. *Experimental Aging Research: An International Journal Devoted to the Scientific Study of the Aging Process, 12 (3),* 169-172.

Strikwerda-Brown, C., Mothakunnel, A., Hodges, J., Piguet, O., & Irish, M. (2019). External details revisited - A new taxonomy for coding 'non-episodic' content during autobiographical memory retrieval. *Journal of Neuropsychology, 13 (3),* 371-397.

Tausczik, Y. R. & Pennebaker, J. W. (2010). The psychological meaning of words: LIWC and computerized text analysis methods. *Journal of Language and Social Psychology, 29 (1),* 24-54.

Uzer, T., & Brown, N. R. (2017). The effect of cue content on retrieval from autobiographical memory. *Acta Psychologica, 172,* 84-91.

Supporting Information

Wheeler, M. A. (2000). A comparison of forgetting rates in older and younger adults. *Aging, Neuropsychology, and Cognition, 7 (3),* 179-193.

Wixted, J. T., & Ebbesen, E. B. (1997). Genuine power curves in forgetting: A quantitative analysis of individual subject forgetting functions. *Memory & Cognition, 25 (5),* 731-739.

Wright, D. B. (1998). Modelling clustered data in autobiographical memory research: The multilevel approach. *Applied Cognitive Psychology, 12,* 339-357.
